# Lineage-specific rediploidization is a mechanism to explain time-lags between genome duplication and evolutionary diversification

**DOI:** 10.1186/s13059-017-1241-z

**Published:** 2017-06-14

**Authors:** Fiona M. Robertson, Manu Kumar Gundappa, Fabian Grammes, Torgeir R. Hvidsten, Anthony K. Redmond, Sigbjørn Lien, Samuel A. M. Martin, Peter W. H. Holland, Simen R. Sandve, Daniel J. Macqueen

**Affiliations:** 1 Institute of Biological and Environmental Sciences, University of Aberdeen, Aberdeen, AB24 2TZ UK; 2 Centre for Integrative Genetics (CIGENE), Faculty of Biosciences, Norwegian University of Life Sciences, Ås, NO-1432 Norway; 3 Department of Chemistry, Biotechnology and Food Science, Norwegian University of Life Sciences, 1432 Ås, Norway; 4 Umeå Plant Science Centre, Department of Plant Physiology, Umeå Plant Science Centre, Umeå University, SE-90187 Umeå, Sweden; 5 Centre for Genome-Enabled Biology & Medicine, University of Aberdeen, Aberdeen, AB24 2TZ UK; 6 Department of Zoology, University of Oxford, South Parks Road, Oxford, OX1 3PS UK

**Keywords:** Whole genome duplication, Rediploidization, Species radiation, Lineage-specific Ohnologue Resolution (LORe), Duplicate genes, Functional divergence, Autotetraploidization, Salmonid fish

## Abstract

**Background:**

The functional divergence of duplicate genes (ohnologues) retained from whole genome duplication (WGD) is thought to promote evolutionary diversification. However, species radiation and phenotypic diversification are often temporally separated from WGD. Salmonid fish, whose ancestor underwent WGD by autotetraploidization ~95 million years ago, fit such a ‘time-lag’ model of post-WGD radiation, which occurred alongside a major delay in the rediploidization process. Here we propose a model, ‘lineage-specific ohnologue resolution’ (LORe), to address the consequences of delayed rediploidization. Under LORe, speciation precedes rediploidization, allowing independent ohnologue divergence in sister lineages sharing an ancestral WGD event.

**Results:**

Using cross-species sequence capture, phylogenomics and genome-wide analyses of ohnologue expression divergence, we demonstrate the major impact of LORe on salmonid evolution. One-quarter of each salmonid genome, harbouring at least 4550 ohnologues, has evolved under LORe, with rediploidization and functional divergence occurring on multiple independent occasions >50 million years post-WGD. We demonstrate the existence and regulatory divergence of many LORe ohnologues with functions in lineage-specific physiological adaptations that potentially facilitated salmonid species radiation. We show that LORe ohnologues are enriched for different functions than ‘older’ ohnologues that began diverging in the salmonid ancestor.

**Conclusions:**

LORe has unappreciated significance as a nested component of post-WGD divergence that impacts the functional properties of genes, whilst providing ohnologues available solely for lineage-specific adaptation. Under LORe, which is predicted following many WGD events, the functional outcomes of WGD need not appear ‘explosively’, but can arise gradually over tens of millions of years, promoting lineage-specific diversification regimes under prevailing ecological pressures.

**Electronic supplementary material:**

The online version of this article (doi:10.1186/s13059-017-1241-z) contains supplementary material, which is available to authorized users.

## Background

Whole genome duplication (WGD) has occurred repeatedly during the evolution of vertebrates, plants, fungi and other eukaryotes (reviewed in [1–4]). The prevailing view is that despite arising at high frequency, WGD is rarely maintained over macroevolutionary (i.e. millions of years (Myr)) timescales, but that, nonetheless, ancient WGD events are over-represented in several species-rich lineages, pointing to a role in long-term evolutionary success [1, 5]. WGD events provide an important source of duplicate genes (ohnologues) with the potential to diverge in protein functions and regulation during evolution [6, 7]. In contrast to the duplication of a single or small number of genes, WGD events are unique in allowing the balanced divergence of whole networks of ohnologues. This is thought to promote molecular and phenotypic complexity through the biased retention and diversification of interactive signalling pathways, particularly those regulating development [8–10].

As WGD events dramatically reshape opportunities for genomic and functional evolution, it is not surprising that an extensive body of literature has sought to identify causal associations between WGD and key episodes of evolutionary history, for example species radiations. Such arguments are clearly appealing and have been constructed for WGD events ancestral to vertebrates [11–15], teleost fishes [16–19] and angiosperms (flowering plants) [10, 20–22]. Nonetheless, it is now apparent that the evolutionary role of WGD is complex, often lineage-dependent and without a fixed set of rules. For example, some ancient lineages that experienced WGD events never underwent radiations, including horseshoe crabs [23] and paddlefish (e.g. [24]), while other clades radiated explosively immediately post-WGD, for example the ciliate *Paramecium* species complex [25]. In addition, apparent robust associations between WGD and the rapid evolution of species or phenotypic-level complexity may disappear when extinct lineages are considered, as proposed for WGDs in the stem of vertebrate and teleost evolution [26, 27].

Such findings either imply that the causative link between WGD and species radiations is weak, or demand alternative explanations. In the latter respect, it is has become evident that post-WGD species radiations may commonly arise following extensive time-lags. For example, major purported species radiations occurred >200 Myr after a WGD in the teleost ancestor (‘Ts3R’) ~320–350 million years ago (Ma) [3, 28, 29]. In angiosperms, similar findings have been reported in multiple clades [30, 31]. Such findings led to the proposal of a ‘WGD Radiation Lag-Time’ model, where some, but not all, lineages within a group sharing ancestral WGD diversified millions of years post-WGD, due to an interaction between a functional product of WGD (e.g. a novel trait) and lineage-specific ecological factors [30]. Within vertebrates, salmonids provide a textbook case of delayed species radiation following an ancestral WGD event ~95 Ma (‘Ss4R’), where a role for ecological factors has been implied [32]. In this respect, salmonid diversification was strongly associated with climatic cooling and the evolution of a life-history strategy called anadromy [32] that required physiological adaptations (e.g. in osmoregulation [33]) enabling migration between fresh and seawater. Importantly, a convincing role for WGD in such cases of delayed post-WGD radiation is yet to be demonstrated, weakening hypothesized links between WGD and evolutionary success. Critically missing in the hypothesized link between WGD and species radiations is a plausible mechanism that constrains the functional outcomes of WGD from arising for millions or tens of millions of years after the original duplication event. Here we provide such a mechanism and uncover its potential impacts on adaptation.

Following all WGD events, the evolution of new molecular functions with the potential to influence long-term diversification processes depends on the physical divergence of ohnologue sequences. This is fundamentally governed by the meiotic pairing outcomes of duplicated chromosomes during the cytogenetic phase of post-WGD rediploidization [11, 34, 35]. Depending on the initial mechanism of WGD, rediploidization can be resolved rapidly or protracted in time. For example, after WGD by allotetraploidization, as recently described in the frog *Xenopus leavis* [36], WGD follows a hybridization of two species and recovers sexual incompatibility [11]. The outcome is two ‘sub-genomes’ within one nucleus that segregate into bivalents during meiosis [35]. In other words, rediploidization is resolved instantly, leaving ohnologues within the sub-genomes free to diverge as independent units at the onset of WGD. The other major mechanism of WGD, autotetraploidization, involves a spontaneous doubling of exactly the same genome. In this case, four identical chromosome sets will initially pair randomly during meiosis, leading to genetic exchanges (i.e. recombination) that prohibit the evolution of divergent ohnologues and enable an ongoing ‘tetrasomic’ inheritance of four alleles [35]. Crucially, rediploidization may occur gradually over tens of millions of years after autotetraploidization [35, 37].

Salmonid fish provide a vertebrate paradigm for delayed rediploidization post-autotetraploidization (reviewed in [37]). The recent sequencing of the Atlantic salmon (*Salmo salar* L.) genome revealed that rediploidization was delayed for one-quarter of the duplicated genome and associated with major genomic reorganizations such as chromosome fusions, fissions, deletions or inversions [38]. In addition, large regions of salmonid genomes still behave in a tetraploid manner in extant species (e.g. [38–40]), despite the passage of ~95 Myr since the Ss4R WGD [32]. In light of our understanding of salmonid phylogeny [32, 41], we can also be certain that rediploidization has been ongoing throughout salmonid evolution [38] and was likely occurring in parallel to lineage-specific radiations [32, 42]. However, the outcomes of delayed rediploidization on genomic and functional evolution remain uncharacterized in salmonids and other taxa. In the context of the commonly reported time-lag between WGD events and species radiation, this represents a major knowledge gap. Specifically, as explained above, a delay in the rediploidization process will cause a delay in ohnologue functional divergence, theoretically allowing functional consequences of WGD to be realized long after the original duplication.

**Fig. 1:**
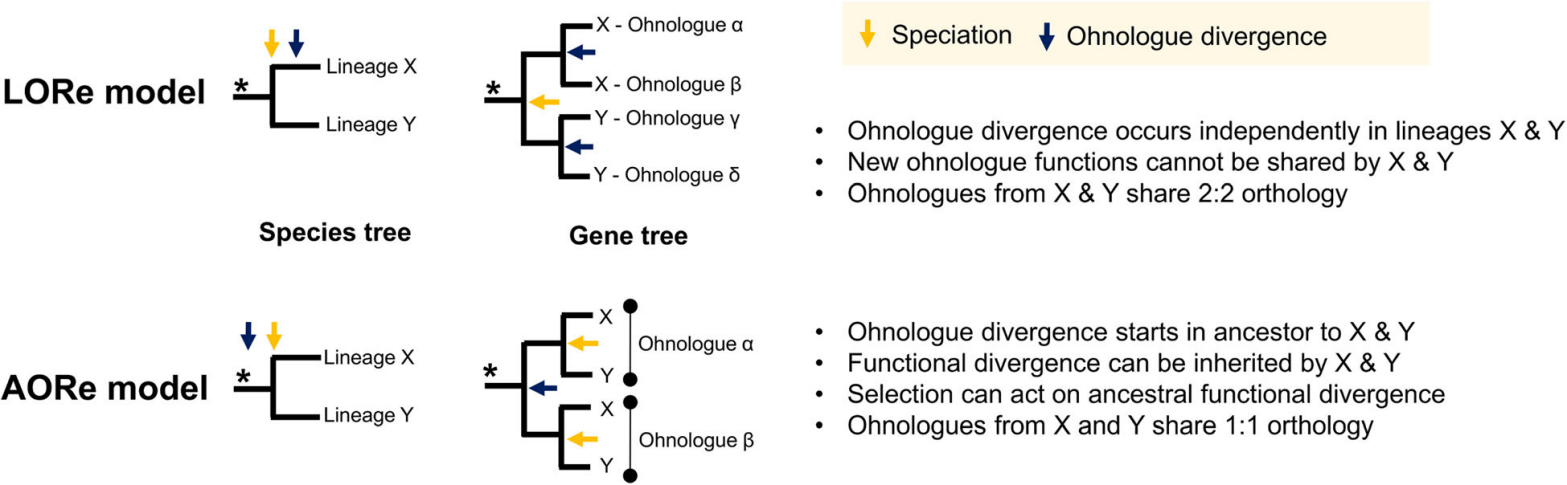
The LORe model of post-WGD evolution following delayed rediploidization. This figure describes the phylogenetic predictions of LORe in contrast to the AORe model, as well as associated implications for functional divergence and sequence homology relationships

Here we propose ‘Lineage-specific Ohnologue Resolution’ or ‘LORe’ as a mechanism to address the role of delayed rediploidization on the evolution of sister lineages sharing an ancestral WGD event (Fig. 1). It builds on and unifies ideas/data presented by Macqueen and Johnston [32], Martin and Holland [43] and Lien et al. [38] and is a logical outcome when rediploidization and speciation events occur in parallel. Under LORe, the rediploidization process is not completed until after a speciation event, which will result in the independent divergence of ohnologues in sister lineages (Fig. 1). This leads to unique predictions compared to the alternative scenario, where ohnologues began to diverge in the ancestor to sister lineages due to ancestral rediploidization (hereafter the ‘Ancestral Ohnologue Resolution’, or ‘AORe’ model). Under LORe, the evolutionary mechanisms allowing functional divergence of gene duplicates [6, 7, 11] become activated independently under lineage-specific selective pressures (Fig. 1). Conversely, under AORe, ohnologues share ancestral selection pressures, which hypothetically increases the chance that similar gene functions will be conserved in different lineages by selection (Fig. 1). A phylogenetic implication of LORe is a lack of 1:1 orthology between ohnologue pairs from different lineages (Fig. 1), leading to the definition of the term ‘tetralog’ to describe a 2:2 homology relationship between ohnologues in sister lineages [43]. Thus, LORe may be mistaken for small-scale duplication if the underlying mechanisms are not appreciated. Despite this, LORe ohnologues have unique phylogenetic properties (Additional file 1: Figure S1) and are distinguished from small-scale gene duplication by their location within duplicated (or ‘homeologous’) blocks on distinct chromosomes sharing collinearity [38, 44, 45].

In this study, we demonstrate that one-quarter of retained salmonid ohnologues (conservatively, 4550 unique genes) have evolved under LORe, which has had a major impact on salmonid fish evolution at multiple levels of genomic and functional organization. Our findings allow us to propose that LORe offers a more broadly applicable mechanism to explain time-lags between many WGD events and subsequent lineage-specific diversification regimes.

## Results

### Extensive LORe followed the Ss4R WGD

**Fig. 2:**
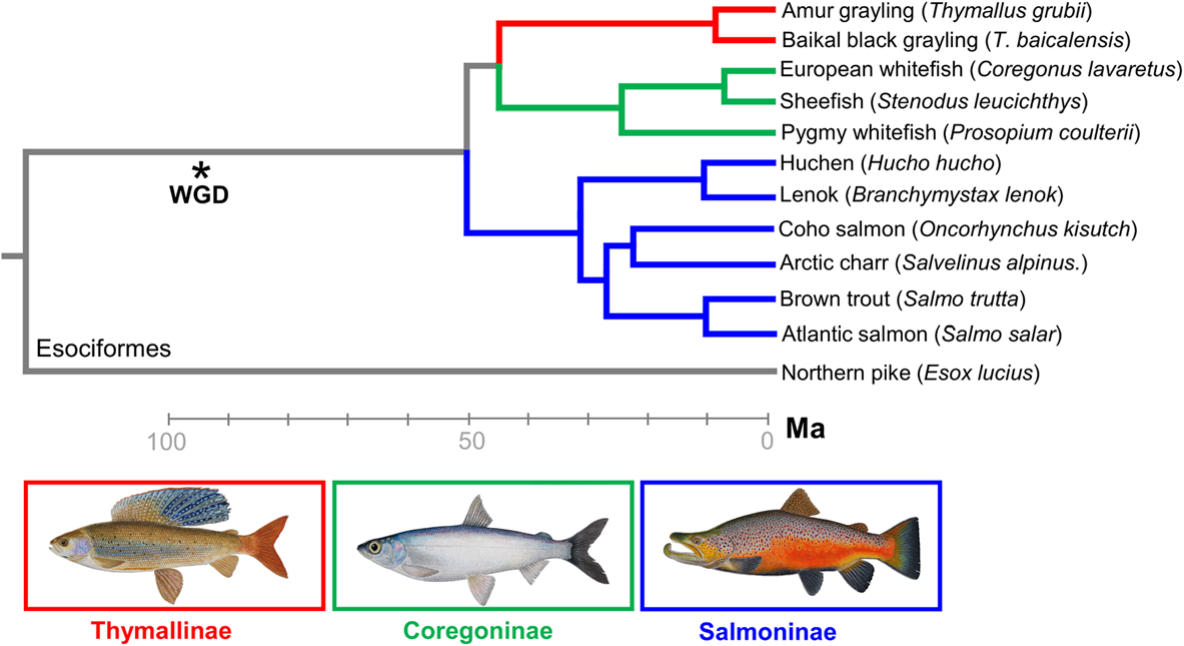
Time-calibrated salmonid phylogeny (after [32]) including the major lineages used for sequence capture and phylogenomic analyses of ohnologues

**Fig. 3:**
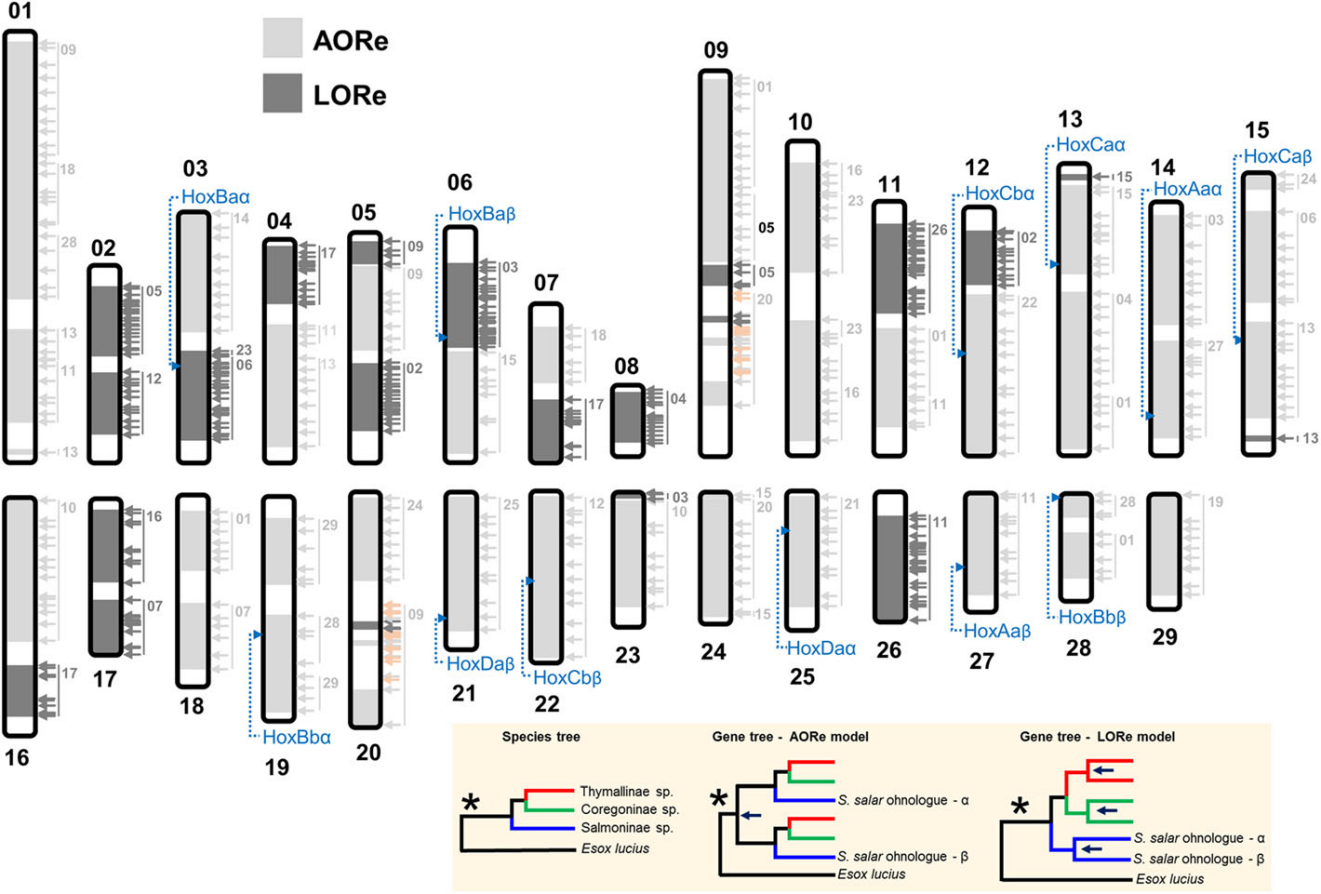
Genome-wide validation of LORe in salmonids. Atlantic salmon chromosomes with LORe and AORe regions of the genome are highlighted, based on sampling 383 separate ohnologue trees (data in Additional file 2). Each *arrow* shows a sampled ohnologue tree (*light grey*, AORe; *dark grey*, LORe; *orange*, ambiguous; Additional file 1: Text S2). The other chromosome in a pair of collinear duplicated blocks [38] is highlighted, along with the genomic location of salmonid Hox clusters. The *shaded* box shows the phylogenetic topologies used to draw conclusions about the LORe versus AORe model in contrast to other scenarios (Additional file 1: Figure S1)

To understand the extent and dynamics of lineage-specific rediploidization in salmonids, we used in-solution sequence capture [46] to generate a genome-wide ohnologue dataset spanning the salmonid phylogeny [32, 41]. Note, here we use the term ohnologue, but elsewhere ‘homeologue’ has been used to describe gene duplicates retained from the Ss4R WGD event [38]. In total, 383 gene trees were analysed (sum of aligned sequence data, 155,166 bp; mean/standard deviation (SD) alignment length, 405/208 bp), sampling every Atlantic salmon chromosome continuously at regular intervals and including ohnologues from at least seven species spanning all the major salmonid lineages plus a sister species (northern pike, *Esox lucius*) that did not undergo the Ss4R WGD [47] (Additional file 2). All the gene trees included verified Atlantic salmon ohnologues based on their location within duplicated (homeologous) blocks sharing common rediploidization histories [38]. Salmonids are split into three subfamilies, Salmoninae (salmon, trout, charr, taimen/huchen and lenok spp.), Thymallinae (grayling spp.) and Coregoninae (whitefish spp.), which diverged rapidly between ~45 and 55 Ma (Fig. 2). Hence, phylogenetic signals of LORe are evidenced by subfamily-specific ohnologue clades (Fig. 1; Additional file 1: Figure S1). In accordance with this, our analysis revealed a consistent phylogenetic signal shared by large continuous duplicated blocks of the genome, with 97% of trees fitting predictions of either the LORe (*n* = 151 trees) or the AORe (*n* = 219 trees) model (Fig. 3; Additional file 2; Additional file 1: Text S2). This finding demonstrates a strong phylogenetic signal of either LORe or AORe, irrespective of the relatively short alignment length that was possible using our sequence capture approach.

The LORe regions defined by phylogenomic analysis represent around one-quarter of the genome and overlap fully with seven pairs of chromosome arms (homeologous arms ‘2p–5q’, ‘2q–12qa’, ‘3q–6p’, ‘4p–8q’, ‘7q–17qb’, ‘11qa–26’ and ‘16qb–17qa’, according to Atlantic salmon nomenclature [38]) known to have undergone delayed rediploidization [38]. Lien et al. [38] reported that each of these chromosome arms shares a higher similarity among ohnologous sequences compared to the rest of the duplicated genome. Our gene tree sampling also revealed two additional, relatively small LORe regions (Fig. 3). The rest of the genome fits to the AORe model in our analysis (Fig. 3) and overlaps fully with collinear blocks located on chromosomes previously concluded to have experienced rediploidization in the salmonid ancestor [38]. Considering the near perfect congruence between our definitions of LORe and AORe and data presented in Lien et al. [38], we can robustly extrapolate that, among 16,786 high-confidence ohnologues identified within genomic regions covered by our analysis (see “Methods”), 27.1% (4550 genes) and 72.9% (12,236 genes) evolved under LORe and AORe, respectively.

**Fig. 4:**
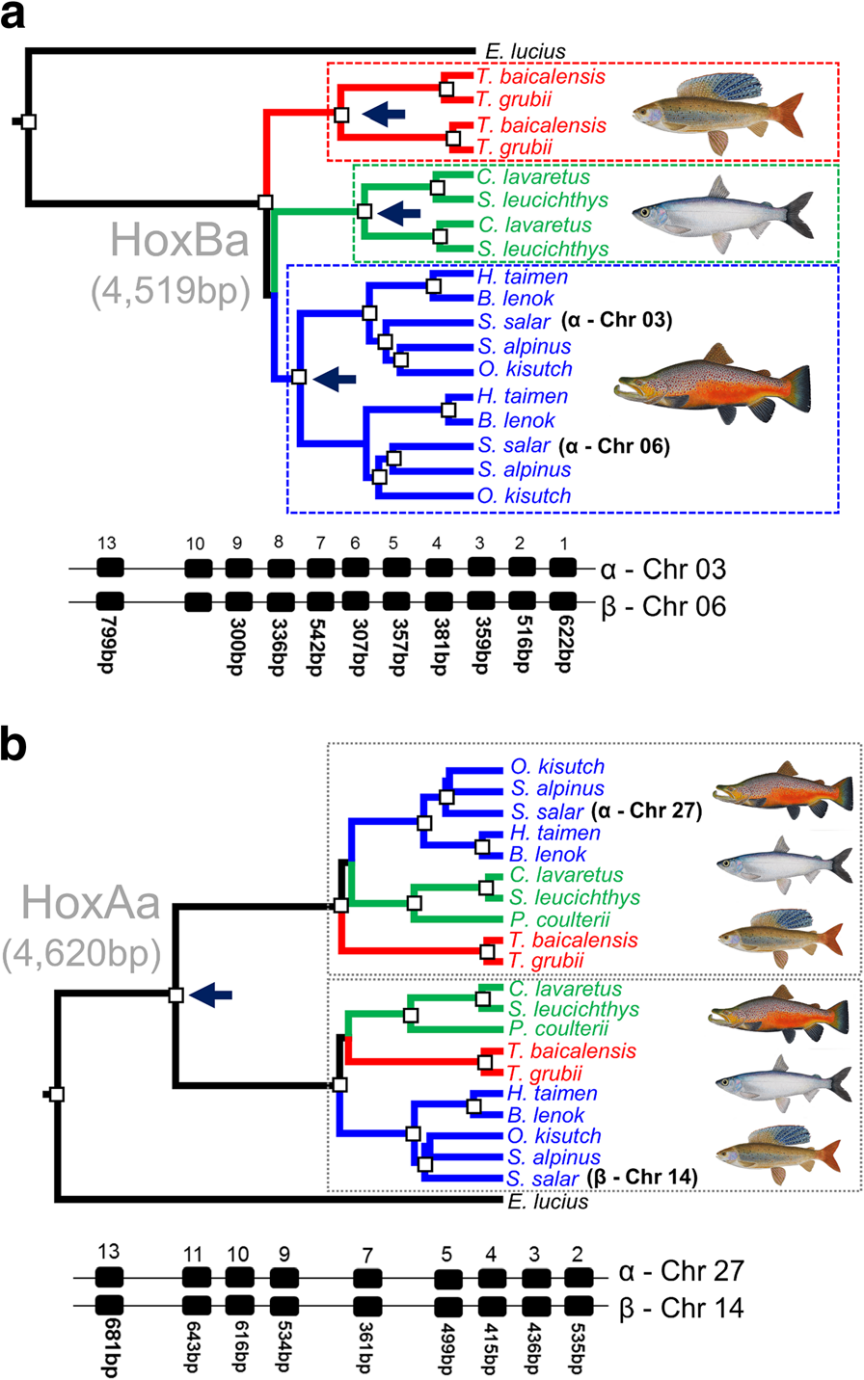
Bayesian phylogenetic analyses of salmonid Hox gene clusters fitting to the predictions of the LORe (**a**) and AORe (**b**) models. *White boxes* depict posterior probability values >0.95. Hox clusters characterized from Atlantic salmon [49] are shown, along with the length of individual sequence alignments combined for analysis. The individual gene trees for Hox alignments are shown in Additional file 1: Figures S2 and S4 for HoxAa and HoxBa, respectively. *Dark blue arrows* highlight the inferred onset of ohnologue divergence, i.e. the node where rediploidization was resolved

To complement our genome-wide overview, we performed a finer-resolution phylogenetic analysis of Hox genes included in our sequence capture study. Hox genes are organized into genomic clusters located across multiple chromosomes and have been used to confirm separate WGD events in the stem of the vertebrate, teleost and salmonid lineages [43, 48, 49]. Phylogenetic analyses of Hox clusters (HoxBa) residing within predicted LORe regions in Atlantic salmon (Fig. 3) strongly supported the LORe model, considering either individual gene trees within a duplicated Hox cluster or trees built from combining separate ohnologue alignments sampled within clusters (e.g. Fig. 4a; Additional file 1: Text S1 and Figures S2–S10). Our data indicate that two salmonid-specific Hox cluster pairs underwent rediploidization as single units, either once independently in the common ancestor of each salmonid subfamily for HoxBa (Fig. 4a) or twice in Coregoninae for HoxAb (Additional file 1: Figure S9 and Text S1). These results cannot be explained by small-scale gene duplication events under any plausible scenario (Additional file 1: Text S1). Thus, HoxAb and HoxBa clusters were in regions of the genome that remained tetraploid until after the major salmonid lineages diverged ~50 Ma (Fig. 2). Phylogenetic analyses of the HoxAa, HoxBb, HoxCa, HoxCb and HoxDa cluster pairs strongly supported the AORe model (e.g. Fig. 4b; Additional file 1: Figure S10 and Text S1), as predicted by genomic location (Fig. 3).

We also studied proteins encoded within Hox clusters to contrast patterns of sequence divergence under the AORe and LORe models (Additional file 1: Figures S11 and S12). As our phylogenetic reconstructions were performed with nucleotide data, we wanted to rule out the possibility that the underlying sequence changes were predominantly synonymous, with little impact at the functional level. The data support our predictions (Fig. 1), as LORe has allowed many amino acid replacements to become independently fixed among Hox ohnologues within each salmonid subfamily (Additional file 1: Figure S12). These changes are typically highly conserved across species, suggesting lineage-specific purifying selection within a subfamily (Additional file 1: Figure S12). Conversely, under the AORe model, numerous amino acid replacements that distinguish Hox ohnologues arose in the common salmonid ancestor and have been conserved across all the major salmonid lineages (Additional file 1: Figure S11).

### Distinct rediploidization dynamics across salmonid lineages

Our data also reveal distinct temporal dynamics of rediploidization across different salmonid lineages. First, using a Bayesian approach, the onset of divergence for the HoxBa-α and -β clusters of Salmoninae, Coregoninae and Thymallinae (i.e. Fig. 4a tree) was estimated at ~46, 25 and 34 Ma (posterior mean values), respectively (95% posterior density intervals of 36–57, 15–37 and 21–47 Ma, respectively). While the confidence intervals on these estimates overlap, the major difference in the mean posterior estimates is consistent with a scenario where the genomic regions containing these Hox clusters experienced rediploidization at substantially different times for the major salmonid lineages.

**Fig. 5:**
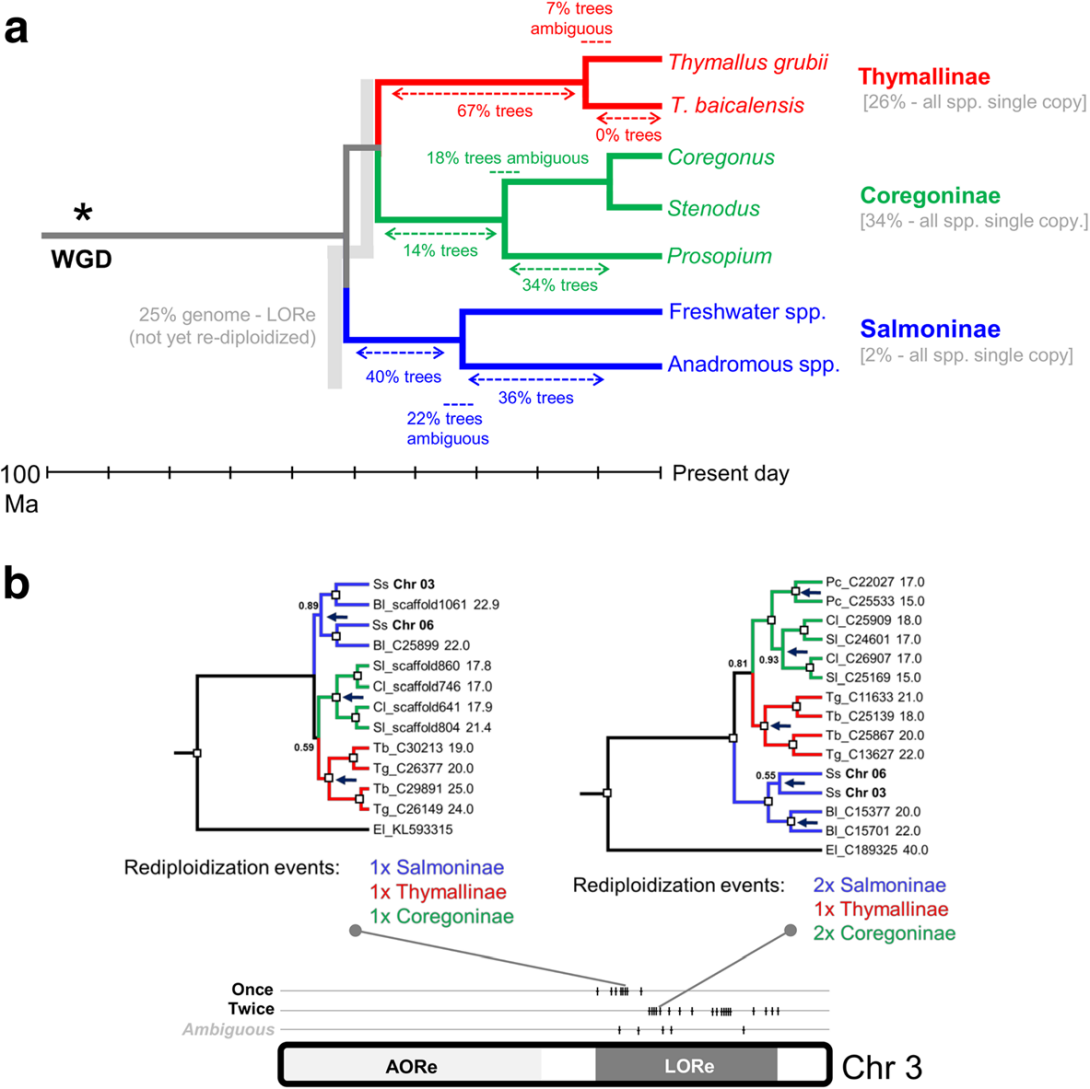
Divergent rediploidization dynamics in different salmonid lineages. **a** Time-tree of species relationships [32] showing the fraction of 383 gene trees supporting independent rediploidization events at different nodes. **b** LORe region on chromosome 03 (paired with an ohnologous region on chromosome 06), where the number of independent rediploidization events inferred within Salmoninae (shown) is consistent along contiguous regions of the genome. Example trees are shown for genomic regions with distinct rediploidization histories. Abbreviations: *Ss S. salar*, *Bl Brachymystax lenok*, *Sl Stenodus leucichthys*, *Cl Coregonus lavaretus*, *Pc Prosopium coulterii*, *Tb Thymallus baicalensis*, *Tg T. grubii*

Further evidence of divergent rediploidization dynamics among salmonid lineages was observed through gene tree sampling (Fig. 3; Additional file 2), which allowed the number of inferred rediploidization events to be mapped along a time-calibrated salmonid phylogeny [32] (Fig. 5a). This was done by recording the divergence between ohnologue pairs (i.e. inferred onset of rediploidization) within each salmonid subfamily in all LORe trees sampled across the genome (*n* = 151; Additional file 2). In Salmoninae, 60/151 (40%) of the sampled genes trees indicated that rediploidization was completed in the stem of this subfamily, before the radiation of extant lineages (Fig. 5a). Assuming 4550 LORe genes (i.e. 2275 ohnologue pairs) underwent rediploidization during Salmoninae evolution, as estimated for the Atlantic salmon genome (i.e. 27.1% of 16,786 genes; see above), and that the Salmoninae stem branch is 19.5 Myr long [32] (Fig. 5a), we extrapolate that ~47 ohnologue pairs underwent rediploidization per Myr (i.e. 40% of 2275 ohnologue pairs/19.5 Myr) during the initial stages of Salmoninae evolution, leading up to the point when anadromy evolved [42]. In contrast, for the whitefish lineage, only 14% of the same LORe gene trees indicated that rediploidization was complete in the stem of extant lineages (Fig. 5a). Assuming the same number of ohnologue pairs were present in the whitefish ancestor and that the relevant stem branch is 25.5 Myr long [32] (Fig. 5a), we extrapolate that ~12 ohnologue pairs underwent rediploidization per Myr (i.e. 14% of 2275 ohnologue pairs/25.5 Myr) in the early stages of whitefish evolution, a rate four times lower than Salmoninae. It is impossible to estimate the rediploidization rate during the equivalent early stages of grayling evolution, as extant lineages diverged within the last 15 Myr [50]. Nonetheless, our data indicate that two-thirds of LORe ohnologues experienced rediploidization in the common ancestor to extant grayling spp. (Fig. 5a).

Interestingly, one-third of all sampled gene trees included a single ohnologue copy for whitefish and grayling, which were clustered along chromosomes in the genome (Additional file 2). As these regions have experienced delayed rediploidization, this likely reflects the ‘collapse’ of highly similar sequences in the assembly process into single contigs [38], rather than the evolutionary loss of an ohnologue. For two LORe regions with evidence of multiple rediploidization events within a salmonid subfamily, we mapped our findings back to Atlantic salmon chromosomes (Fig. 5b). This showed that the number of inferred rediploidization events within a LORe region is consistent across large genomic regions (Fig. 5b; Additional file 1: Figure S13). Overall, these data support past observations that the rediploidization process is dependent on chromosomal location [38], while emphasizing distinct dynamics of rediploidization in different salmonid subfamilies.

### Regulatory divergence under LORe

**Fig. 6:**
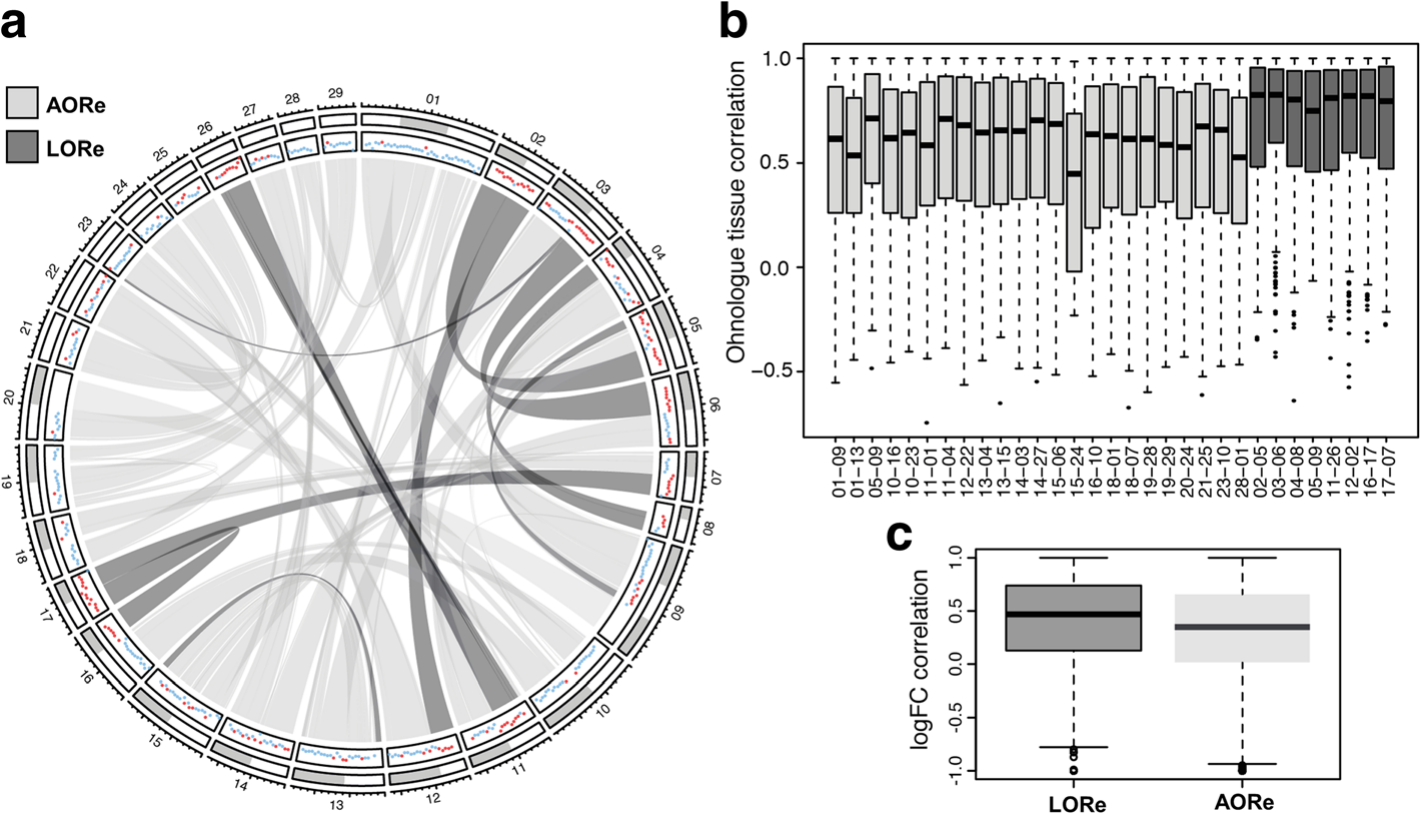
Global consequences of LORe for ohnologue expression evolution. **a** Circos plot of Atlantic salmon chromosomes highlighting LORe and AORe regions defined by phylogenomics. The panel with *coloured dots* indicates expression similarity among ohnologue pairs: each *dot* represents the correlation of ohnologue expression across a 4-Mb window. *Red* and *blue dots* show correlations ≥0.6 and <0.6, respectively. **b** Correlation in expression levels across 15 tissues for ohnologue pairs in AORe and LORe regions. Different collinear blocks are shown [38] containing at least ten ohnologue pairs. **c** The overall correlation in the expression responses of ohnologues from LORe and AORe regions (2505 and 6853 pairs, respectively) during the physiological transition from fresh to saltwater. The correlation was calculated for log fold-change responses across nine tissues

To better understand the functional implications of LORe, we contrasted the level of expression divergence between Atlantic salmon ohnologue pairs from AORe and LORe regions (Fig. 6). This was done in multiple tissues under controlled conditions (Fig. 5a, b) and also following ‘smoltification’ [33], a physiological remodelling that accompanies the life-history transition from freshwater to saltwater in anadromous salmonid lineages (Fig. 6c). In regions of the genome covered by our analysis, ohnologue expression was more correlated within LORe than AORe regions, both across tissues (Fig. 6b; Wilcoxon test, *P* = 2.2e-16) and considering differences in regulation between fresh and saltwater (Fig. 6c; Wilcoxon test, *P* = 5.1e-10). A recent analysis [38] suggested that 28% of salmonid ohnologues fit a model of expression divergence where one duplicate maintained the ancestral tissue expression (as observed in northern pike) and the other acquired a new expression pattern (i.e. ‘regulatory neofunctionalization’ [38]). We extended this analyses by partitioning ohnologue pairs from LORe and AORe regions of the genome. Among 2021 ohnologue pairs displaying regulatory neofunctionalization, ~19 versus ~81% were located in LORe and AORe regions, respectively, constituting a significant enrichment in AORe regions compared to the background expectation (i.e. 27.1 versus 72.9%; hypergeometric test, *P* = 2e-13). The average higher correlation in expression and lesser extent of regulatory neofunctionalization for ohnologues in LORe regions is expected, as they have had less evolutionary time to diverge in terms of sequences controlling mRNA-level regulation. Nonetheless, many ohnologues in LORe regions have diverged in expression (Fig. 6), which may have contributed to phenotypic variation available solely for lineage-specific adaptation.

### Role of LORe in lineage-specific evolutionary adaptation

To better understand the role of LORe in adaptation, we performed an in-depth analysis of Atlantic salmon genes with established or predicted functions in smoltification [33], which we hypothesize represent important factors for the lineage-specific evolution of anadromy. Interestingly, LORe regions contain ohnologues for many genes from master hormonal systems regulating smoltification, including the insulin-like growth factor (IGF), growth hormone (GH), thyroid hormone (TH) and cortisol pathways (Additional file 1: Table S1) [33, 51–53]. Notably, the actual master hormones from the IGF and GH pathways, i.e. encoding IGF1 and GH, which are together crucial for the development of seawater tolerance [33, 51], represent LORe ohnologues. We also identified many LORe ohnologues within a large set of genes involved in osmoregulation and cellular ionic homeostasis, key for saltwater tolerance, including Na+, K+-ATPases (targets for the above mentioned hormones [33, 51]), along with members of the ATP-binding cassette transporter, solute carrier and carbonic anhydrase families (Additional file 1: Table S1). Several additional genes from the same systems were represented by ohnologues in AORe regions (Additional file 1: Table S1).

**Fig. 7:**
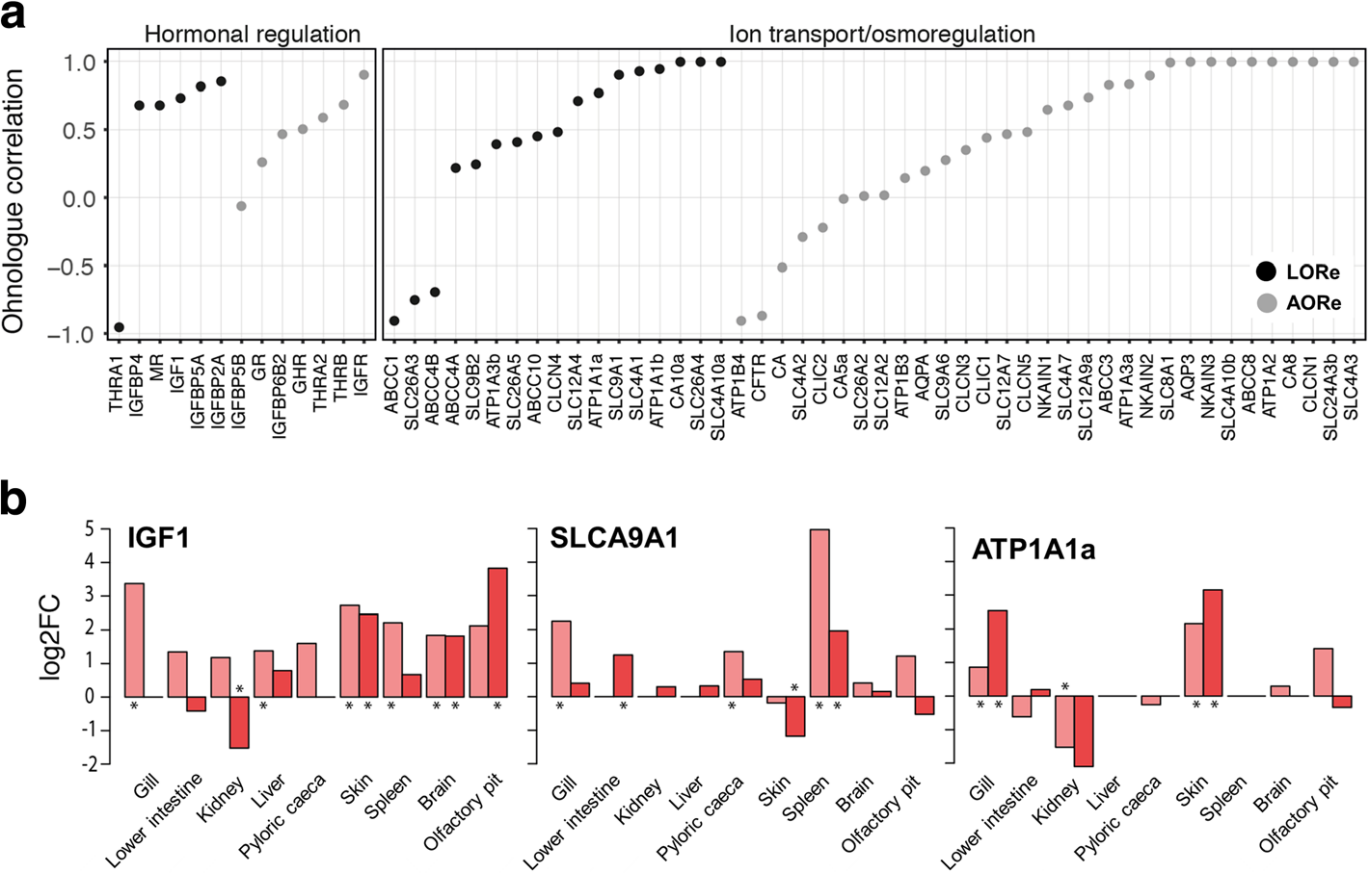
Regulatory evolution of salmonid ohnologues implied in anadromy defined within a lineage-specific context of physiological adaptation. **a** Correlation in expression responses for ohnologues from LORe versus AORe regions during the fresh to saltwater transition in Atlantic salmon. Each name on the *x-axis* is a pair of ohnologues (details in Additional file 1: Table S1). The data are ordered from the most to least correlated ohnologue expression responses. Correlation was performed using Pearson’s method. Data for additional ohnologues where correlation was impossible due to a restriction of expression to a limited set of tissues are provided in Additional file 3. **b** Example ohnologues showing a multi-tissue differential expression response to the fresh to saltwater transition. The *asterisks* highlight significant expression responses. Equivalent plots for all genes shown in **a** are provided in Additional file 3

To characterize the regulatory evolution of ohnologues with roles in smoltification, we compared equivalent tissue expression ‘atlases’ from Atlantic salmon in fresh and saltwater (Fig. 7; Additional file 3). The extent of regulatory divergence was variable for ohnologues in both LORe and AORe regions, ranging from conserved to unrelated tissue responses (Fig. 7a; Additional file 3). Several pairs of ohnologues from both LORe and AORe regions showed marked expression divergence in tissues of established importance for smoltification (examples in Fig. 7b; Additional file 3). For example, a pair of LORe ohnologues encoding IGF1 located on chromosomes 07 and 17 (i.e. homeologous arms 7q–17qb under Atlantic salmon nomenclature [38]), despite differing by only a single conservative amino acid replacement, were differentially regulated in several tissues (Fig. 5b). The differential regulation of IGF1 ohnologues in gill and kidney is especially notable, as both tissues are vital for salt transport and, in gill, this hormone stimulates the development of chloride cells and the upregulation of Na+, K+-ATPases, together required for hypo-osmoregulatory tolerance [54, 55]. Thus, key expression sites for IGF1 are evidently fulfilled by different LORe ohnologues and these divergent roles have evolved specifically within the Salmoninae lineage, 40–50 Myr post WGD [32]. In contrast to IGF1, LORe ohnologues encoding GH showed highly conserved regulation during smoltification (Additional file 3). Overall, these findings demonstrate that many Atlantic salmon ohnologues in both LORe and AORe regions are differentially regulated under a physiological context that recaptures lineage-specific adaptations linked to anadromy.

To further characterize the role of LORe in lineage-specific adaptation, we performed gene ontology (GO) enrichment analysis contrasting all ohnologues present in LORe versus AORe regions (Additional file 4). Remarkably, ohnologues in LORe versus AORe regions were enriched for 99.9% non-overlapping GO terms, suggesting global biases in encoded functions (Additional file 4). The most significantly enriched GO terms for LORe ohnologues were ‘indolalkylamine biosynthesis’ and ‘indolalkylamine metabolism’ (Additional file 4). This is notable as 5-hydroxytryptamine is an indolalkylamine and the precursor to serotonin, which plays an important role controlling the master pituitary hormones that govern smoltification [51, 56]. An interesting feature of rediploidization is the possibility that functionally related genes residing in close genomic proximity (e.g. due to past tandem duplication) started diverging into distinct ohnologues as single units, for example Hox clusters (Fig. 4). We found that LORe ohnologues contributing to enriched GO terms ranged from being highly clustered in the genome to not at all clustered (Additional file 1: Table S2). In the latter case, we can exclude biases linked to regional rediploidization history. In the former case, we noted that two clusters of globin ohnologues on chromosomes 03 and 06 (i.e. homeologous arms ‘3q-6p’ under Atlantic salmon nomenclature [38]) explain the enriched term ‘oxygen transport’ (Additional file 1: Table S2). This is interesting in the context of lineage-specific adaptation, as haemoglobin subtypes are regulated during smoltification to increase oxygen-carrying capacity and meet the higher aerobic demands of the oceanic migratory phase of the life-cycle [33]. Other GO terms enriched for LORe ohnologues included pathways regulating growth and protein synthesis, immunity, muscle development, proteasome assembly and the regulation of oxidative stress and cellular organization (Additional file 4).

## Discussion

Here we define the LORe model and characterize its impacts on multiple levels of organization, adding a novel layer of complexity to our understanding of evolution after WGD. While past analyses have highlighted the quantitative extent of delayed rediploidization for a single salmonid genome [38], our study is the first to establish the genome-wide functional impacts of LORe and is unique in revealing divergent long-term rediploidization dynamics across the major salmonid lineages. Our results show that salmonid ohnologues can have strikingly distinct evolutionary ‘ages’, both for different genes located within the same genome (Figs. 3 and 4) and when comparing the same genes in phylogenetic sister lineages sharing the same ancestral WGD (Fig. 5). Our data also indicate that thousands of LORe ohnologues have diverged in regulation or gained novel expression patterns tens of Myr after WGD, likely contributing to lineage-specific phenotypes (Fig. 7). Hence, in the presence of highly delayed rediploidization, all ohnologues are not ‘born equal’ and many will have opportunities to functionally diverge under unique environmental and ecological contexts, for example during different phases of Earth’s climatic and biological evolution in the context of salmonid evolution [32]. It is also notable that ohnologues retained in LORe and AORe regions of the genome are enriched for different functions (Additional file 4), suggesting unique roles in adaptation, similar to past conclusions gained from comparison of ohnologues versus small-scale gene duplicates (e.g. [57, 58]). However, LORe is quite distinct from small-scale duplication, considering that large blocks of genes with common rediploidization histories will get the chance to diverge in functions in concert, meaning selection on duplicate divergence can operate on a multi-genic level.

LORe is possible whenever speciation precedes (or occurs in concert) to rediploidization (Fig. 1). This scenario is probable whenever rediploidization is delayed, most relevant for autotetraploidization events, which have occurred in plants [59], fungi [2] and unicellular eukaryotes (e.g. [25]) and was the likely mechanism of WGD in the stem vertebrate and teleost lineages [35, 43, 60]. However, LORe is not predicted under a strict definition of allotetraploidization, as cytogenetic rediploidization is resolved immediately. Nonetheless, after some allotetraploidization events, the parental genomes have high regional similarity (i.e. segmental allotetraploidy [61]), allowing prolonged tetrasomic inheritance in some genomic regions, leading to potential for LORe. Interestingly, past studies have provided indirect support for LORe outside salmonids, including following WGD in the teleost ancestor [43]. A recent analysis of duplicated Hox genes from the lamprey *Lethenteron japonicum* failed to provide evidence of 1:1 orthology comparing jawed and jawless vertebrates, leading to the radical suggestion of independent, rather than ancestral vertebrate WGD events [62]. However, if rediploidization was delayed until after the divergence of these major vertebrate clades, which occurred no more than 60–100 Myr after the common vertebrate ancestor split from ‘unduplicated’ chordates [63, 64], such findings are parsimoniously explained by LORe. In other words, WGD events may be shared by all vertebrates [60, 65], but some ohnologues became diploid independently in jawed and jawless lineages. Gaining unequivocal support for LORe beyond salmonids will require careful phylogenomic approaches akin to those employed here.

Our findings also reveal a possible mechanism to explain why some lineages experienced delayed post-WGD species radiations, i.e. the WGD radiation lag-time model [30–32]. This is a topical subject, given the recent suggestion that teleosts radiated at a similar rate to their sister lineage (holosteans) in the immediate wake of the teleost-specific WGD (Ts3R) [27], but nonetheless experienced later radiations [28, 29]. Our results suggest that, in the presence of delayed rediploidization, the functional outcomes of WGD need not arise ‘explosively’, but can be mechanistically delayed for tens of Myr. For example, tissue expression responses for master genes required for saltwater tolerance are evidently fulfilled by one member of a salmonid ohnologue pair that first began to diverge in functions 40–50 Myr post-WGD (Fig. 7). Hence, in light of evidence for delayed rediploidization after Ts3R [43], an alternative interpretation is that teleosts gained an increasing competitive advantage through time compared to their unduplicated sister group, via the drawn-out creation of functionally divergent ohnologue networks that provided greater scope for adaptation to ongoing environmental change. Similar arguments apply for delayed radiations in angiosperm lineages sharing WGD with a sister clade that diversified at a lower rate [30, 31], offering a worthy area of future investigation.

For salmonids, climatic cooling likely provided a selective pressure promoting the lineage-specific evolution of anadromy, which, according to formal diversification rate tests, facilitated speciation in the long-term [32]. Interestingly, we observed an elevated rediploidization rate in Salmoninae compared to other lineages leading up to the time that anadromy evolved. Taken with the lineage-specific regulatory divergence of LORe ohnologues regulating smoltification (Figs. 5 and 7), we hypothesize that LORe contributed to the evolution of lineage-specific adaptations that promoted species radiation. However, the role of LORe in adaptation is likely complex, occurring in a genomic context where an existing substrate of AORe ohnologues (that have had greater opportunity to diverge in function) can also contribute to lineage-specific adaptation. This is evident in our data, as many relevant ohnologues from AORe regions of the genome show extensive regulatory divergence in the context of smoltification (Fig. 7; Additional file 3). A realistic scenario for lineage-specific adaptation involves functional interactions between networks of newly diverging LORe ohnologues and ‘older’ AORe ohnologues that have already diverged in function from the ancestral state. Nonetheless, even though all ohnologues may undergo lineage-specific functional divergence, only during the initial stages of LORe will neofunctionalization and subfunctionalization [6, 7, 11] arise without the influence of purifying selection on past functional divergence (Fig. 1). In the future, follow-up questions on the roles of both classes of salmonid ohnologues (and indeed other types of gene duplicate) in lineage-specific adaptation will become possible through comparative analysis of multiple salmonid genomes, done in a phylogenetic framework spanning the evolutionary transition to anadromy [66].

## Conclusions

Our results empirically validate the LORe model and demonstrate its unappreciated significance as a nested component of genomic and functional divergence following WGD. LORe should now be considered within future investigations into the role of WGD as a driver of evolutionary adaptation and diversification, including delayed post-WGD radiations.

## Methods

### Target-enrichment and Illumina sequencing

To generate a genome-wide ohnologue set for phylogenomic analyses in salmonids, we used in-solution sequence capture with the Agilent SureSelect platform prior to sequencing on an Illumina HiSeq2000. Full methods were recently detailed elsewhere, including the source and selection of 16 study species [46]. While this past study was a small-scale investigation of a few genes [46], here we up-scaled the approach to 1293 unique capture probes (Additional file 5; Additional file 1: Text S3 provides details on probe design). 120mer oligomer baits were synthesised at fourfold tiling across the full probe set and a total of 1.5 Mbp of unique sequence data were produced in each capture library. The captures were performed on randomly fragmented gDNA libraries, meaning that the recovered data represent exons plus flanking genomic regions [46]. We recovered 21.7 million reads per species on average after filtering low-quality data (SD, 0.8 million reads; >99.1% paired-end data; Additional file 1: Table S3), which were assembled using SOAPdenovo2 [67] with a K-mer value of 91 and merging level of 3 (otherwise default parameters). Species-specific BLAST databases [68] were created for downstream analyses. Assembly statistics were assessed via the QUAST webserver [69] (Additional file 1: Table S3). We used BLAST and mapping approaches to confirm that the sequence capture worked efficiently with high specificity and that pairs of ohnologues had been routinely recovered, even when a single ohnologue was used as a capture probe (full details given in Additional file 1: Text S3).

### Phylogenomic analyses

This work was split into a genome-wide investigation and a detailed study of Hox clusters. For both approaches, sequence data were sampled from our capture databases for different salmonid spp. using BLASTn [68] and aligned with MAFFT v.7 using the default automatic strategy [70]. Northern pike was used as the outgroup to the Ss4R WGD in all analyses; this species was included in our target-enrichment study, but pike sequences were captured slightly less efficiently compared to salmonids [46]. Thus, we supplemented pike sequences using the latest genome assembly [47] (ASM72191v2; NCBI accession CF_000721915). All phylogenetic tests were done at the nucleotide level within the Bayesian Markov chain Monte Carlo (MCMC) framework BEAST v.1.8 [71], specifying an uncorrelated lognormal relaxed molecular clock model [72] and the best-fitting substitution model (inferred by maximum likelihood in Mega v.6.0 [73] for individual alignments and PartitionFinder [74] for combined alignments). The MCMC chain was run for 10-million generations and sampled every 1000th generation. TRACER v.1.6 [75] was used to confirm adequate mixing and convergence of the MCMC chain (effective sample sizes >200 for all estimated parameters). Maximum clade credibility trees were generated in TreeAnnotator v.1.8 [71]. All sequence alignments and Bayesian gene trees are provided in Additional file 2, including details on ohnologues sampled from the Atlantic salmon genome, alignment lengths and the best-fitting substitution model.

For the genome-wide study, the 1293 unique capture probes were used in BLASTn searches against the Atlantic salmon genome (ICSASG_v2; NCBI accession GCF_000233375) via http://salmobase.org/. This provided a genome-wide overview of the location of ohnologue alignments that could be generated via our capture assemblies and confidence that the targeted genes were true ohnologues retained from the Ss4R WGD, based on their location within collinear duplicated (homeologous) blocks [38]. In total, 383 ohnologue alignments were generated, using the appropriate probes as BLAST queries against our capture databases to acquire the sequence data. The selection of gene trees sampled among those available from the sequence capture data was done to maximize the overall representation of each salmon chromosome, with a higher sampling effort performed in putative LORe regions, i.e. chromosome arms with a known history of delayed rediploidization [38]. Each tree contained a pair of verified ohnologues from Atlantic salmon and putative ohnologues captured from at least one species per each of the most distantly related lineages within the three salmonid subfamilies.

For the Hox study, we used 89 Hox genes from Atlantic salmon [53] as BLASTn queries against our capture assemblies. The longest captured regions were aligned, leading to 54 alignments (accounted for within the 383 ohnologue alignments mentioned above) spanning all characterized Hox clusters [53]. We performed individual-level phylogenetic analyses on each dataset, revealing a highly consistent phylogenetic signal across different Hox genes from each Hox cluster (Additional file 1: Figure S2–S8), allowing alignments to be combined to the level of whole Hox clusters. To estimate the timing of rediploidization in the duplicated HoxBa cluster of salmonids [49], we employed the dataset combining all sequence alignments (i.e. tree in Fig. 4a). However, the analysis was done after setting calibration priors at four nodes according to MCMC posterior estimates of divergence times from a previous fossil-calibrated analysis [32]. The calibrations were made for the ancestor to two salmonid-specific HoxBa ohnologue clades for Salmoninae and Coregoninae. For Salmoninae, we set the prior for the common ancestor to *Hucho*, *Brachymystax*, *Salvelinus*, *Salmo* and *Oncorhynchus* (normally distributed, median = 32.5 Ma; SD, 3.5 Ma; 97.5% interval, 25–39 Ma). For Coregoninae, we set the prior for the common ancestor to *Stenodus leucichthys* and *Coregonus lavaretus* (normally distributed, median = 4.2 Ma; SD, 0.9 Ma; 97.5% interval, 2.4–5.7 Ma). We ran the calibrated BEAST analysis without data to confirm the intended priors were recaptured in the MCMC sampling.

### RNAseq analyses

To analyse ohnologue regulatory divergence in an appropriate physiological context to explore the evolution of anadromy, we performed RNAseq on nine Atlantic salmon tissues (gill, lower intestine, kidney, liver, pyloric caeca, skin, spleen, brain, olfactory pit) sampled before and after smoltification (see Additional file 6 for detailed information on samples and mapping statistics). Six fish (three males and three females) were sampled from both freshwater (i.e. pre-smoltification, *n* = 6; mean/SD length, 18.6/0.5 cm) and saltwater (i.e. post-smoltification, *n* = 6; mean/SD length, 25.8/0.8 cm) at AquaGen facilities (Trondheim, Norway). RNA extraction was performed on each tissue and its purity and integrity were assessed using a Nanodrop 1000 spectrophotometer (Thermo-Scientific) and 2100 BioAnalyzer (Agilent), respectively. Subsequently, libraries were produced from 2 μg of total RNA using a TruSeq stranded total RNA sample Kit (Illumina, USA) according to the manufacturer’s instructions (Illumina #15031048 Rev.E). Sequencing was performed on a MiSeq instrument using a v.3 MiSeq Reagent Kit (Illumina) generating 2 × 300 bp, strand-specific, paired-end reads. For each tissue, the sequenced individuals were pooled into two sets of three individuals of each sex in both freshwater and saltwater (hence, any reported responses are common to males and females; sex-specific differences were not considered in this study). For the global analysis of ohnologue expression divergence in different tissues under controlled conditions (i.e. Fig. 6a, b), we employed high-coverage Illumina transcriptome reads previously generated for 15 Atlantic salmon tissues (described in [38]).

In both RNAseq analyses, raw Illumina reads were subjected to adapter and quality trimming using cutadapt [76], followed by quality control with FastQC, before mapping to the RefSeq genome assembly (ICSASG_v2) using STAR v.2.3 [77]. Uniquely mapped reads were counted using the HTSeq python script [78] in combination with a modified RefSeq.gff file. The .gff file was modified to contain the attribute “gene_id” (file accessible at http://salmobase.org/download.html). Expression levels were calculated as counts per million total library counts in EdgeR [79]. Total library sizes were normalised to account for bias in sample composition using the trimmed mean of m-values approach [77]. For the smoltification study, log-fold expression changes were calculated, contrasting samples from freshwater and saltwater, done separately for each tissue using EdgeR [79]. Genes showing a false discovery rate-corrected *P* value ≤0.05 were considered differentially expressed.

To identify salmonid-specific ohnologue pairs in AORe and LORe regions of the Atlantic salmon genome, a self-BLASTp analysis was done using all annotated RefSeq proteins, keeping only proteins coded by genes within verified collinear (homeologous) regions retained from the Ss4R WGD [38] with >50% coverage and >80% identity to both query and hit. Statistical analyses on expression data were performed using various functions within R [80]. Expression divergence was estimated using Pearson correlation in all cases. The Circos plot (Fig. 6a) was generated using the circlize library in R [81].

### GO enrichment analyses

GO annotations for Atlantic salmon protein-coding sequences were obtained using Blast2GO [82]. The longest predicted protein for each gene was blasted against Swiss-Prot (http://www.ebi.ac.uk/uniprot) and processed with default Blast2GO settings [83]. The results have been bundled into an R package (https://gitlab.com/cigene/R/Ssa.RefSeq.db). Protein-coding genes were tested for enrichment of GO terms belonging to the sub-ontology ‘biological process’ using a Fisher test implemented in the Bioconductor package topGO [83]. The analysis was restricted to terms of a level higher than four, with more than 10 but less than 1000 assigned genes. Enrichment analyses were done separately for all ohnologue pairs with annotations retained in LORe (2002 pairs) and AORe (5773 pairs) regions of the RefSeq genome assembly. We recorded the chromosomal locations of LORe ohnologues for the most significantly enriched GO terms, including the number of unique LORe regions they occupy in the genome (Additional file 1: Table S2). The rationale was to establish the extent to which ohnologues underlying an enriched GO term are physically clustered. We devised a ‘clustering index’, quantifying the total number of cases where n ≥ 2 ohnologues present within the relevant genomic regions are located within 500 kb of each other, expressed as a proportion of n − 1 the total number of ohnologues located within those regions. A respective clustering index of 1.0, 0.5 and 0.0 means that all, half or zero of the ohnologues accounting for an enriched GO term are located within 500 kb of their next nearest gene within the same genomic region; 500 kb was considered a conservative distance to capture genes expanded by tandem duplication.

## Additional files

Additional file 1: Supporting text, figures and tables. Contains **Text S1–S3**, **Figures S1–S13** and **Tables S1–S3**. (PDF 3119 kb)

Additional file 2: Phylogenomic analysis. Full data associated with the 383 ohnologue gene trees used to define LORe and AORe regions, including: (i) NCBI accession number and gene/protein details for sequence capture probes, (ii) genomic location of ohnologues in the Atlantic salmon genome, (iii) the length of each sequence alignment, (iv) the substitution model used for Bayesian phylogenetic analysis, (iv) the number of inferred rediploidization events (v), each gene tree (nexus format), and (vi) alignments used to generate each gene tree (fasta format). (XLSX 1984 kb)

Additional file 3: Full ohnologue expression response data (summarized in Fig. 7). Multi-tissue expression responses accompanying the freshwater to saltwater transition for candidate Atlantic salmon ohnologues with implied functions in smoltification and anadromous life-history. (PDF 1844 kb)

Additional file 4: Ohnologue GO enrichment analyses. Full enrichment data for GO terms belonging to the sub-ontology ‘biological process’, done comparing protein-coding ohnologues located in LORe and AORe regions. (XLSX 7524 kb)

Additional file 5: Sequence capture probes. Full data associated with the 1293 sequence probes used to capture a genome-wide dataset of ohnologues across 15 salmonid species, including: (i) NCBI accession and encoded protein product for each sequence capture probe, (ii) the salmonid species from which each probe sequence was taken, (iii) ‘Probe status’, where ‘Singleton’ means only a single gene duplicate was included in the probe set, even if other duplicates were identified and ‘Duplicate’ means both gene duplicates were present in the probe set, and (iv) ‘Selection status’, where ‘Pre-selected’ means the sequence capture probe was selected a priori and ‘Selected randomly’ means the genes were chosen at random. (XLSX 588 kb)

Additional file 6: Detailed RNAseq information. Full data associated with transcriptomic analysis of ohnologue expression responses accompanying the freshwater to saltwater transition in Atlantic salmon, including: (i) sample metadata, (ii) read and mapping statistics, (iii) gene/feature count statistics, and (iv) NCBI SRA accession numbers. (XLSX 18 kb)
