# Supplementary material for: Lineage-specific rediploidization is a mechanism to explain time-lags between genome duplication and evolutionary diversification
Source: Genome Biol. 2017 Jun 14;18:111. doi: 10.1186/s13059-017-1241-z (PMC5470254; doi:10.1186/s13059-017-1241-z)
Supplement: Supplementary file 1 — Supporting text, figures and tables. Contains Text S1–S3, Figures S1–S13 and Tables S1–S3. (PDF 3119 kb) [file 13059_2017_1241_MOESM1_ESM.pdf]

Additional file 1

Lineage-specific rediploidization is a mechanism to explain time-lags between genome duplication and evolutionary diversification

Fiona M. Robertson, Manu Kumar Gundappa, Fabian Grammes, Torgeir R. Hvidsten, Anthony K. Redmond, Sigbjørn Lien, Samuel A.M. Martin, Peter W. H. Holland, Simen R. Sandve & Daniel J. Macqueen

Contents of Additional file 1:

Supporting text: Text S1-S3 & references specific to Additional file 1

Supporting figures: Fig. S1 to S13

Supporting tables: Table S1 to S3

Supporting text:

Text S1: **Phylogenetic analyses of salmonid Hox clusters**

AORe model supported for five salmonid Hox cluster pairs

The Atlantic salmon Hox clusters include six ohnologue pairs retained from the salmonid-specific ('Ss4R') WGD along with one singleton cluster [49]. The nomenclature used with respect to ohnologues retained from the teleost-specific ('Ts3R') and Ss4R WGD events is a/b and α/β, respectively [49]. There was a consistent phylogenetic signal in support of AORe model predictions for multiple salmonid ohnologues of HoxAa (Fig. S2), HoxBb (Fig. S5), HoxCa (Fig. S6), HoxCb (Fig. S7) and HoxDa (Fig. S8). All the individual trees produced for these clusters included two separate salmonid clades, each represented once by each salmonid species and including one of the two salmonid-specific ohnologues from Atlantic salmon, where genomic organization has been established [49]. Relationships within each clade were consistent with robust molecular phylogenies [e.g. 32, 41], suggesting a strong signal of orthology across the captured salmonid ohnologues.

For the salmonid HoxAa, HoxCa and HoxCb duplicate clusters, combining the phylogenetic signal within different sampled alignments provided maximal statistical support (posterior probability 1.0) for the tree root representing the split of the northern pike from salmonids (Fig. S9), even though one fifth of the individual trees placed this species as a sister group to one of the salmonid-specific ohnologue clades, evidently at random along each cluster and with weak support (Fig. S2, S6 and S7). Considering the dominant signal indicating the expected branching of pike, these individual trees likely represent artefacts linked to the short length of individual alignments leading to a violation of the molecular clock. However, for the HoxDa analysis, northern pike was a sister to the salmonid clade containing the Atlantic salmon HoxDaα ohnologue in every individual phylogenetic analysis (n=7) (Fig. S8), which was recaptured in the combined analysis (Fig. S9). This raises the possibility that HoxDaα and HoxDaβ arose before the split of Salmonidae and Esociformes. However, this interpretation requires additional assumptions, including the loss of an entire HoxDa cluster in northern pike and cannot easily explain the detectable absence of salmonid-specific ohnologues for HoxDaα and HoxDaβ. Therefore, the consistent branching of pike with HoxDaα may represent an artefact linked to regional genomic differences in the pattern of evolution between the HoxDa clusters, again causing a violation of the relaxed molecular clock model.

LORe supported for two Hox cluster pairs

Phylogenetic analyses including 10 Atlantic salmon ohnologue pairs from the HoxBa cluster produced radically different topologies to those fitting the AORe model (Fig. S4). In an analysis combining the phylogenetic signal of each of these sampled alignments, the three salmonid subfamilies were monophyletic and independently split into two sister clades, each represented by the breadth of study species (posterior probability: 1.0; see Fig. 4A). Moreover, northern pike was maximally supported as the sister branch to all salmonids (Fig. 4A). This topology matches to predictions of LORe, as described in Fig. 1 and Fig. S1, assuming that permanent ohnologue divergence started independently within the basal evolution of each of the three salmonid subfamilies. An alternative scenario enforcing the AORe model requires the loss of a salmonid-specific Hox cluster in the ancestor to salmonids, followed by a minimum of three independent small-scale duplication events (assuming doubling of entire clusters; small-scale duplications of single Hox genes would require a much larger number of events), one in each subfamily (see Fig. S1). This scenario is extremely unlikely for vertebrate Hox genes, where local duplication events within a cluster are yet to be observed and all expansions have occurred via WGD.

Phylogenetic analyses including five Atlantic salmon genes spanning the 'singleton' HoxAb cluster led to trees consistent with predictions of LORe (Fig. S3 and S9). In this case, we repeatedly identified a single orthologue of each Atlantic salmon HoxAb gene in all other members of Salmoninae and exactly two unique sequences in species from Coregoninae and Thymallinae. The presence of a single HoxBa cluster in Salmoninae can be explained by the loss of one ohnologous cluster in the common subfamily ancestor or alternatively, may reflect a region where rediploidization has yet to be resolved, or was resolved so recently that little ohnologue divergence has evolved, leading the assembly process to collapse into single contigs [38]. There was evidence that LORe of HoxAb occurred twice within Coregoninae, separately within *Prosopium* and *Coregonus* lineages (Fig. S3; Fig. S9), which was commonly observed in our genome-wide phylogenetic analyses (Fig. 5).

Text S2: **Ambiguous trees in the genome-wide LORe analysis**

As evidenced in Fig. 3 and Additional file 1, our genome-wide sampling of phylogenetic trees was almost always accompanied by a strong phylogenetic signal along verified duplicated collinear blocks of the genome, with only 13 out of 383 trees having an ambiguous topology out-with predictions of the LORe or AORe model. Interestingly, these trees were not randomly distributed, and concentrated within a single duplicated block maintaining collinearity across chromosomes 9 and 20 (or '9qc–20qb' using Atlantic salmon nomenclature [38]). We sampled 23 trees from the 9qc–20qb region (Additional file 1), of which 3 and 7 fit predictions of LORe and the AORe model, respectively (the remaining 13 being the ambiguous trees) (Additional file 1). This is notable, as 9qc–20qb is the only region in the genome where we observed AORe and LORe trees physically interspersed within a single duplicated collinear block. Inspection of the ambiguous trees failed to reveal consistent branching patterns to explain why 9qc–20qb is an outlier in our analysis. Instead, the branching patterns included a range of paraphyletic groupings, involving different subfamilies and their ohnologues that could not be reconciled with either LORe or the AORe model. 9qc–20qb is known to be unusual in maintaining an average level of similarity mid-way between regions of the duplicated genome that unambiguously match predictions of LORe vs. the AORe model (Fig. 3) [38]. However, we cannot currently explain our findings without unwarranted speculation. Nonetheless, this results points to unique rediploidization dynamics underlying the divergence of 9qc–20qb compared to the remaining genome. A high-resolution comparative analysis of salmonid genomes will be needed to further address this puzzle.

Text S3. **Further details on the sequence capture study**

Design of capture baits

Additional file 5 includes the sequences and accession numbers for 1,293 unique capture probes used in our study. The probes represented cDNAs mainly encoding complete protein sequences. The probes were from several salmonid species, predominantly Atlantic salmon *Salmo salar* (1,024 probes), rainbow trout *Oncorhynchus mykiss* (160 probes) and coho salmon *Oncorhynchus kisutch* (99 probes). Approximately 40% (514) of the probes were pre-selected to cover functional pathways of prior interest. These genes were extracted by BLASTn, either against NCBI or transcriptome databases for *O. mykiss* [Supplemental ref 1] and *O. kisutch* [Supplemental ref 2]. In Additional file 6, sequences obtained from transcriptome databases have been assigned an accession number for a closely related sequence (>99% identity) from *S. salar* or *O. mykiss*. 60% (776) of the probes were randomly selected *S. salar* genes. 69% (893) of the probes represented 'singleton' genes, where the sequences of any potential gene duplicates were absent from the probe set, even when such duplicates existed. The remaining 400 sequences (31% of probes) represented putative salmonid-specific ohnologue pairs/groups defined from past work or via BLAST analyses (see section below).

Efficiency of sequence capture

The efficiency of sequence capture across different levels of probe-to-target sequence divergence was calculated by mapping raw reads captured from northern pike (*Esox lucius*) back to the pike genome [51] (Fig. S14). This was done only for sequences with a 1:1 relationship between the salmonid probe and target pike gene. Therefore, the analysis was restricted to the top BLASTn hits in the pike genome (>80% sequence identity cut-off) for each of the 893 singleton probes. This removed any confounding effects arising from the presence of two ohnologue probes within the sequence capture mix. The Burrows-Wheeler Aligner (BWA) - MEM algorithm [Supplemental ref 3] was used for mapping pike reads back to the pike genome and the average coverage of mapped regions was calculated in Geneious v. 9 [Supplemental ref 4]. The probe-to-target percentage sequence identity was calculated based on alignment of the relevant pike genes to the relevant probe sequence. Pearson's correlation was calculated in SPSS v 22 (IBM Corp. Released 2013. IBM SPSS Statistics for Windows, Version 22.0. Armonk, NY: IBM Corp.), comparing the probe-to-target percentage sequence identity versus the mean mapped coverage of captured reads. This approach confirmed that sequence capture worked efficiently across large genetic distances (Fig. S14), spanning probe-to-target nucleotide identities of 72 to 97% (average 88.3% divergence; SD: 3.7%). The returned coverage across different probes ranged from 9x to 2,333x (average: 374x coverage; SD: 264x). There was a significant, but weak predictive effect of probe-to-target nucleotide identity on the efficiency of the sequence capture (R =7.1%, P <0.0001). Thus, using salmonid probes to capture pike genes was highly effective across a large sample of different genes and divergence levels.

Assessing the capture of salmonid ohnologues

Species-specific assemblies of the captured reads were used in reciprocal BLAST searches against the singleton probes in order to estimate the proportion of putative gene duplicates captured by single probe sequences for all salmonid species (Fig. S15). BLAST searches were conducted using local BLAST v2.4.0+ [68]. The top 5 hits in each assembly with sequence similarity >85% to the probe sequence across at least 100bp (1e-0.20 cut-off) were assessed to determine if the probe sequence matched to one or two unique sequences across the length of captured regions corresponding to the probe sequence. When >1 sequence was recovered by a singleton probe sequence, they were defined as being unique if they shared <98% identity, which is the upper end of similarity between salmonid-specific ohnologues [38]. Moreover, a lower cut-off of 85% sequence identity was selected, as this is the lower end of sequence identity between salmonid-specific ohnologue pairs within salmonid genomes [38]. Of the 893 singleton probes queried, on average 99.5% (904 probes, SD=0.2%) returned contigs representative of at least one gene copy over the 15 salmonid species (Fig. S15). Around half of all genes are retained as ohnologues from the Ss4R WGD [e.g. 38, 45] and our sequence capture data fall in line with these expectations, as 45.1% (SD = 3.3%) of the BLAST searches of singleton probes captured two paralogous genes (Fig. S15). Finally, 100% of assembled contigs for one randomly selected species (*Brachymystax lenok*) had a significant BLAST hit against the original probes (1e-0.20 cut-off),

indicating that the assemblies were also highly specific to the original probes. Taken with our past work [46] and data published within this paper, it is clear that the Agilent sureselect platform provides a highly repeatable approach to obtain recently duplicated genes from any salmonid species, including salmonid-specific ohnologues, even when only one gene duplicate is present in the capture probe set.

Supplemental References

1. Macqueen DJ, Garcia de la Serrana D, Johnston IA. Evolution of ancient functions in the vertebrate insulin-like growth factor system uncovered by study of duplicated salmonid fish genomes. Mol Biol Evol. 2013;30:1060-76.

2. Kim JH, Leong JS, Koop BF, Devlin RH. Multi-tissue transcriptome profiles for coho salmon (Oncorhynchus kisutch), a species undergoing rediploidization following whole-genome duplication. Mar Genomics. 2016;25:33-7.

3. Li H, Durbin R. Fast and accurate short read alignment with Burrows-Wheeler transform. Bioinformatics. 2009;25:1754-60.

4. Kearse M, Moir R, Wilson A, Stones-Havas S, Cheung M, Sturrock S, Buxton S, Cooper A, et al. Geneious Basic: an integrated and extendable desktop software platform for the organization and analysis of sequence data. Bioinformatics. 2012;28:1647-9.

Supporting Figures:

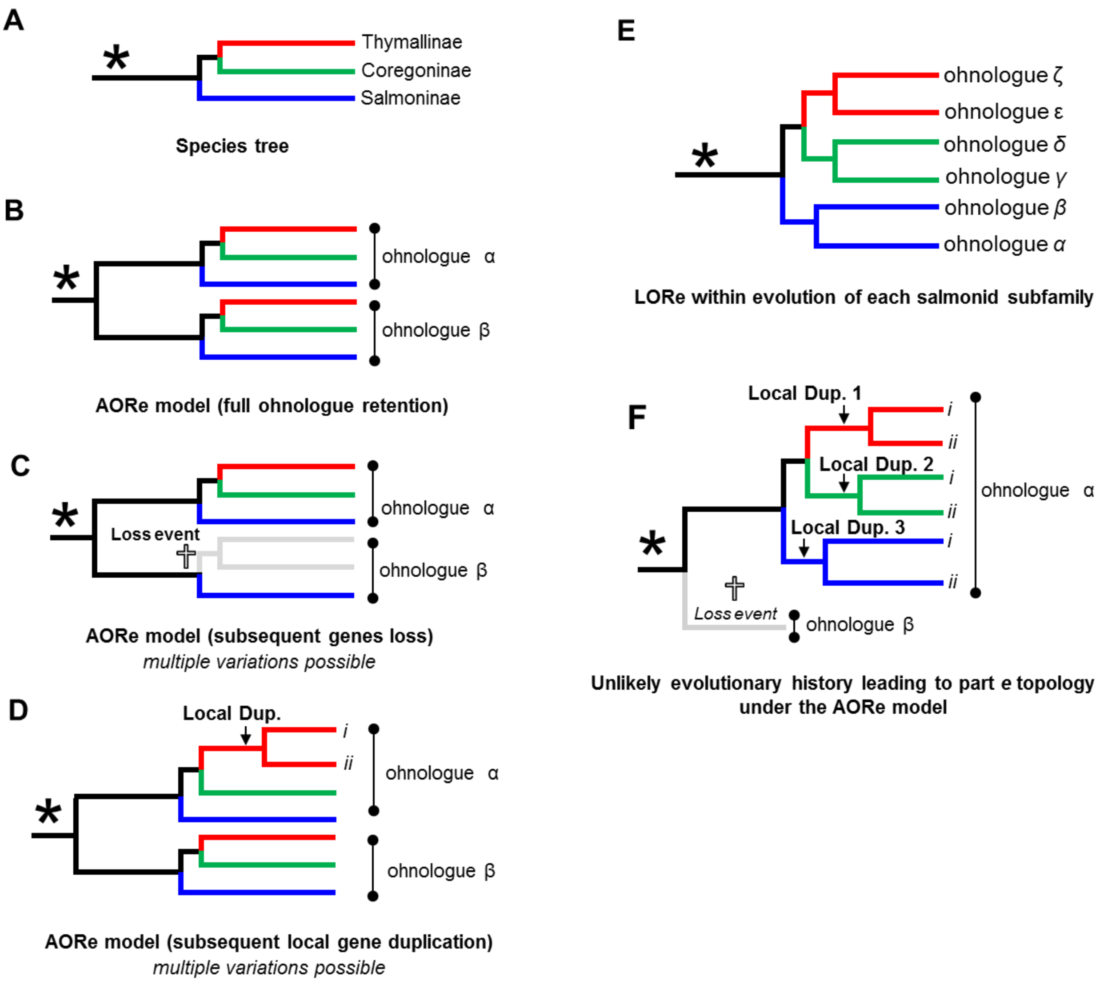

Fig. S1. Phylogenetic predictions of LORe vs. the AORe model following Ss4R WGD. (**A**) Phylogenetic relationships of the three salmonid subfamilies. (**B**) Topology expected under the AORe model, with ohnologue divergence starting in the salmonid ancestor. (**C** & **D**) Respective topologies predicted under the AORe model involving additional gene losses or local gene duplication events. **E**) Topology expected if LORe occurred within the evolution of each salmonid subfamily. The inclusion of multiple lineages within each salmonid subfamily enable more precise inference of the point where ohnologue divergence (i.e. rediploidization) started. **F**) Hypothetical evolutionary scenario where the part **E** tree is expected under the AORe model. This scenario requires the loss of one salmonid-specific ohnologue ancestrally followed by multiple independent local duplications in different salmonid lineages. Importantly, in our genome-wide analyses (Fig. 3), we always included verified salmonid-specific ohnologues defined independently by their location in collinear blocks retained from the Ss4R WGD [38]. This step adds confidence that the part **E** topology is not a product of small-scale duplication.

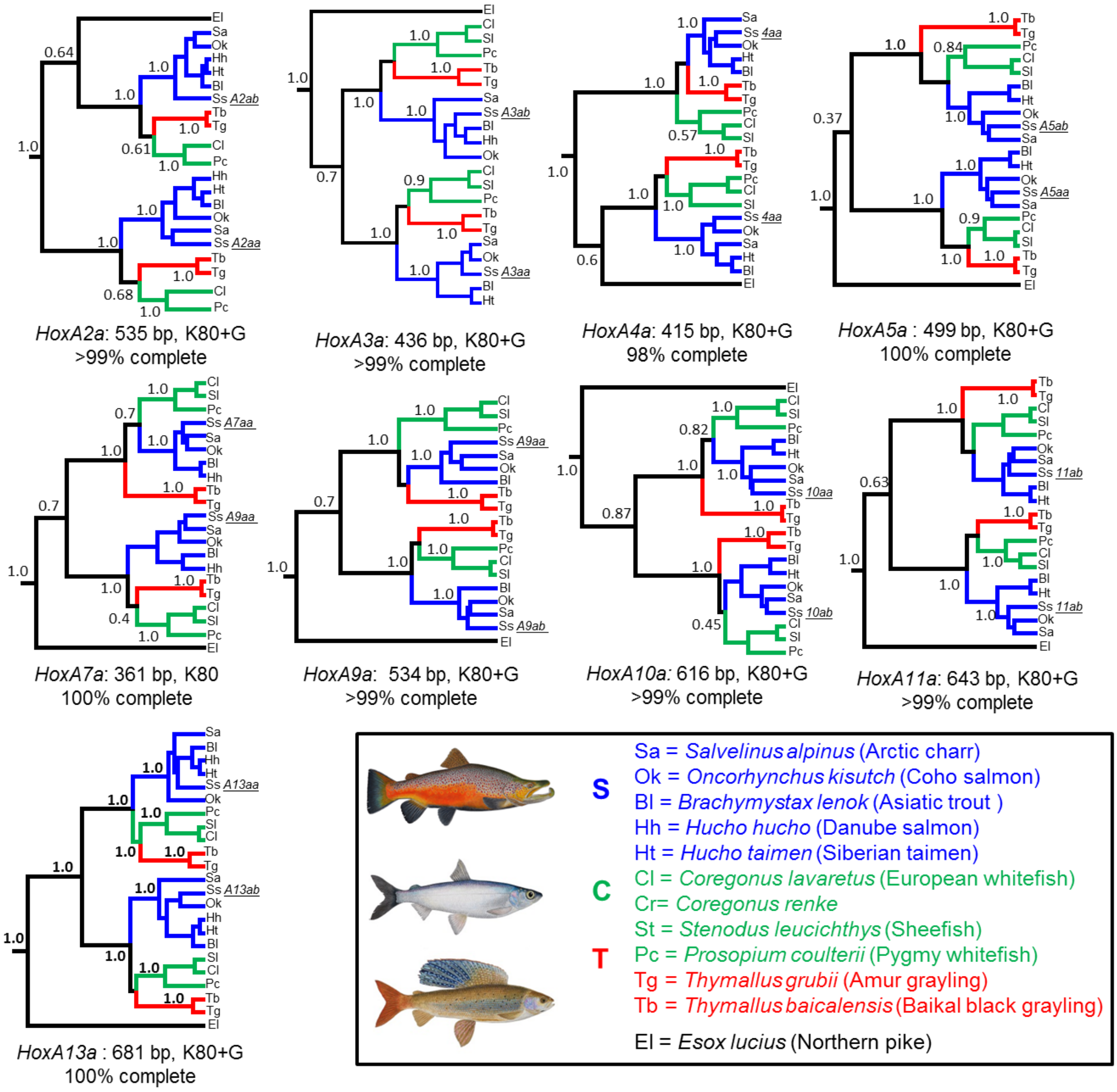

Fig. S2. Phylogenomic analysis of salmonid-specific HoxAa clusters. Individual Bayesian phylogenetic trees are shown for the sequence capture alignments combined in Fig. 3B (main text). Each analysis incorporated a relaxed molecular clock model and the best-fitting nucleotide substitution model (shown). The length of each alignment and average completeness of included sequences is shown. Each tree includes posterior probability values for every node leading up to the crown of salmonid subfamilies. A key to taxa names and branch colouring is provided. Alignments and trees are provided within Additional file 1.

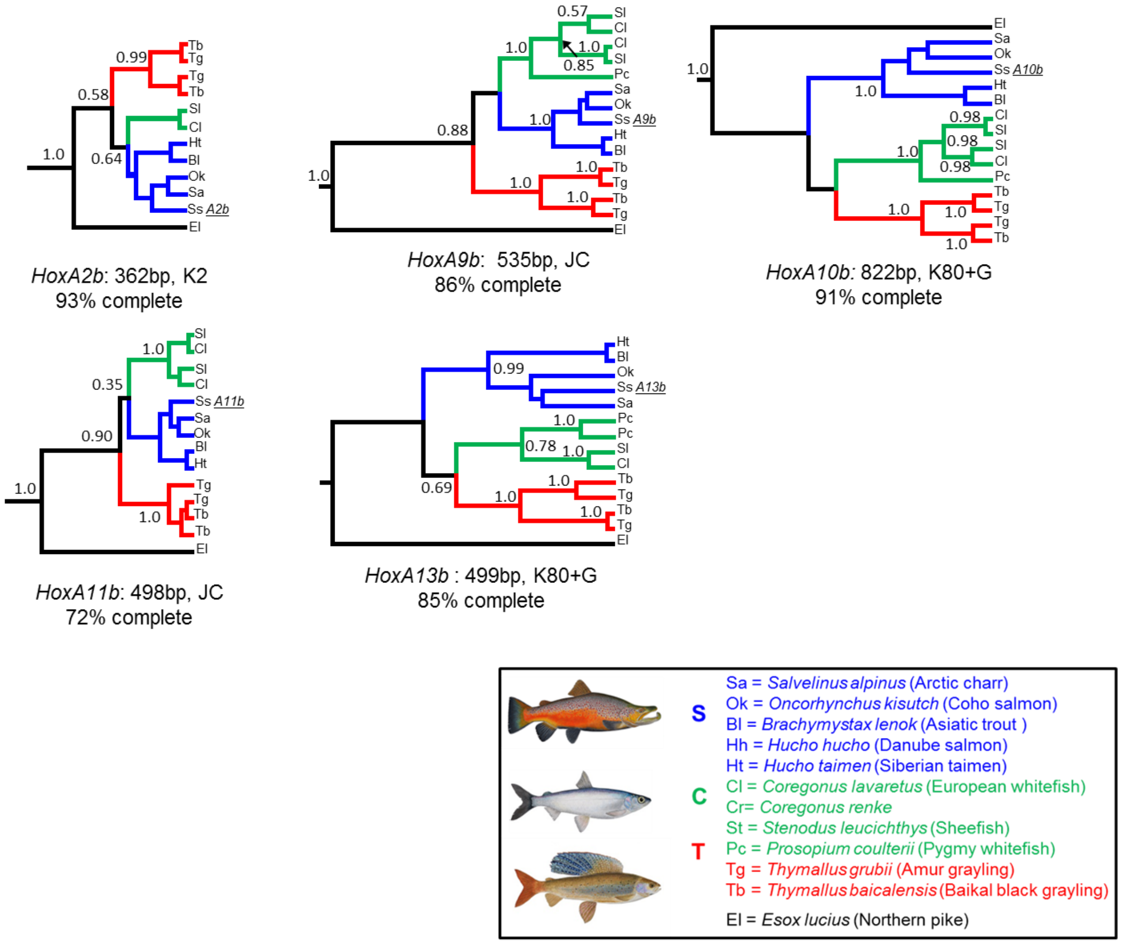

Fig. S3. Phylogenomic analysis of salmonid-specific HoxAb clusters. Individual Bayesian phylogenetic trees are shown for the sequence capture alignments combined for use in Fig. S10. All other details are as provided in the Fig. S2 legend.

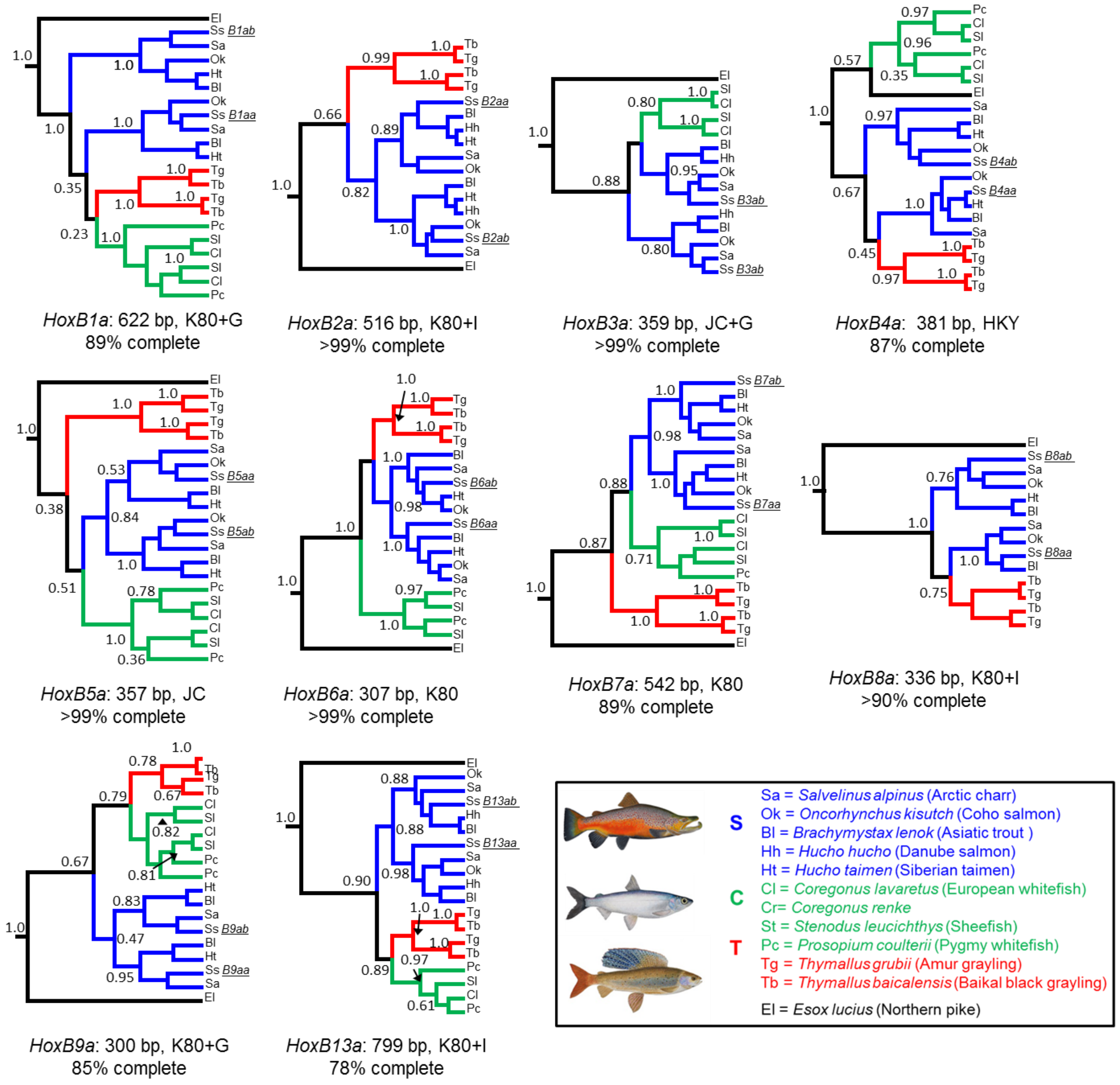

Fig. S4. Phylogenomic analysis of salmonid-specific HoxBa clusters. Individual Bayesian phylogenetic trees are shown for the sequence capture alignments combined for use in Fig. 3B (main text). All other details are as provided in the Fig. S2 legend.

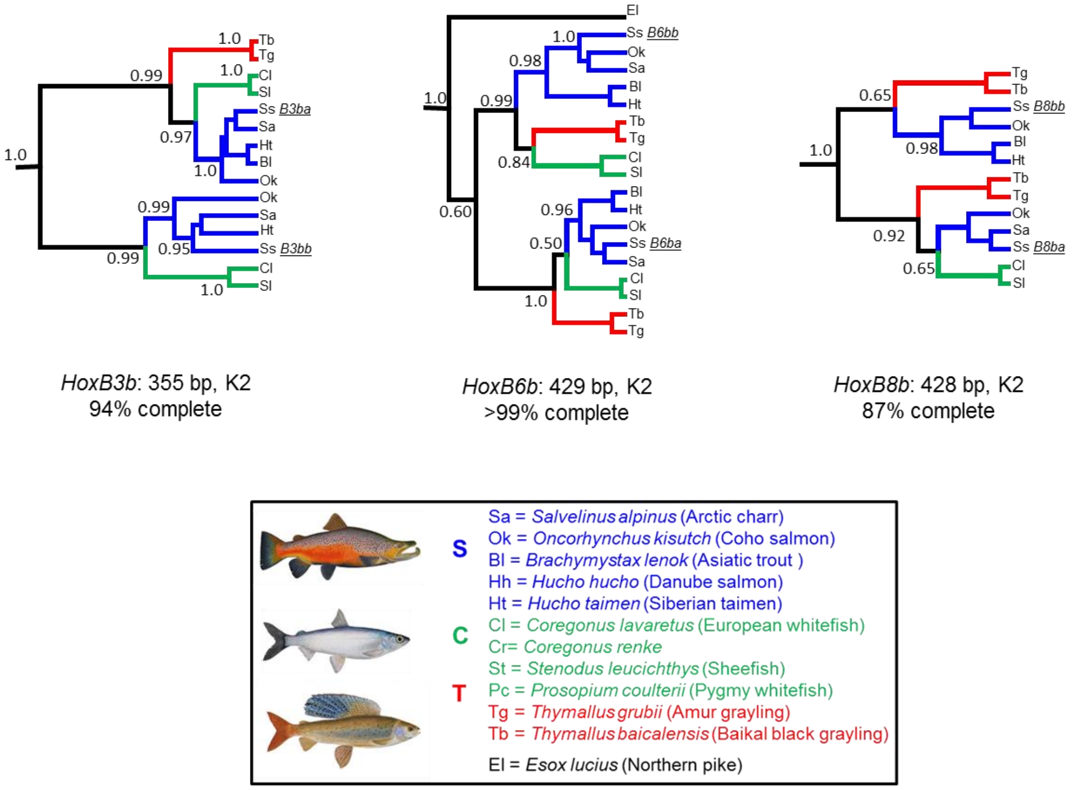

Fig. S5. Phylogenomic analysis of salmonid-specific HoxBb clusters. Individual Bayesian phylogenetic trees are shown for the sequence capture alignments combined for use in Fig. S9A. All other details are as provided in the Fig. S2 legend.

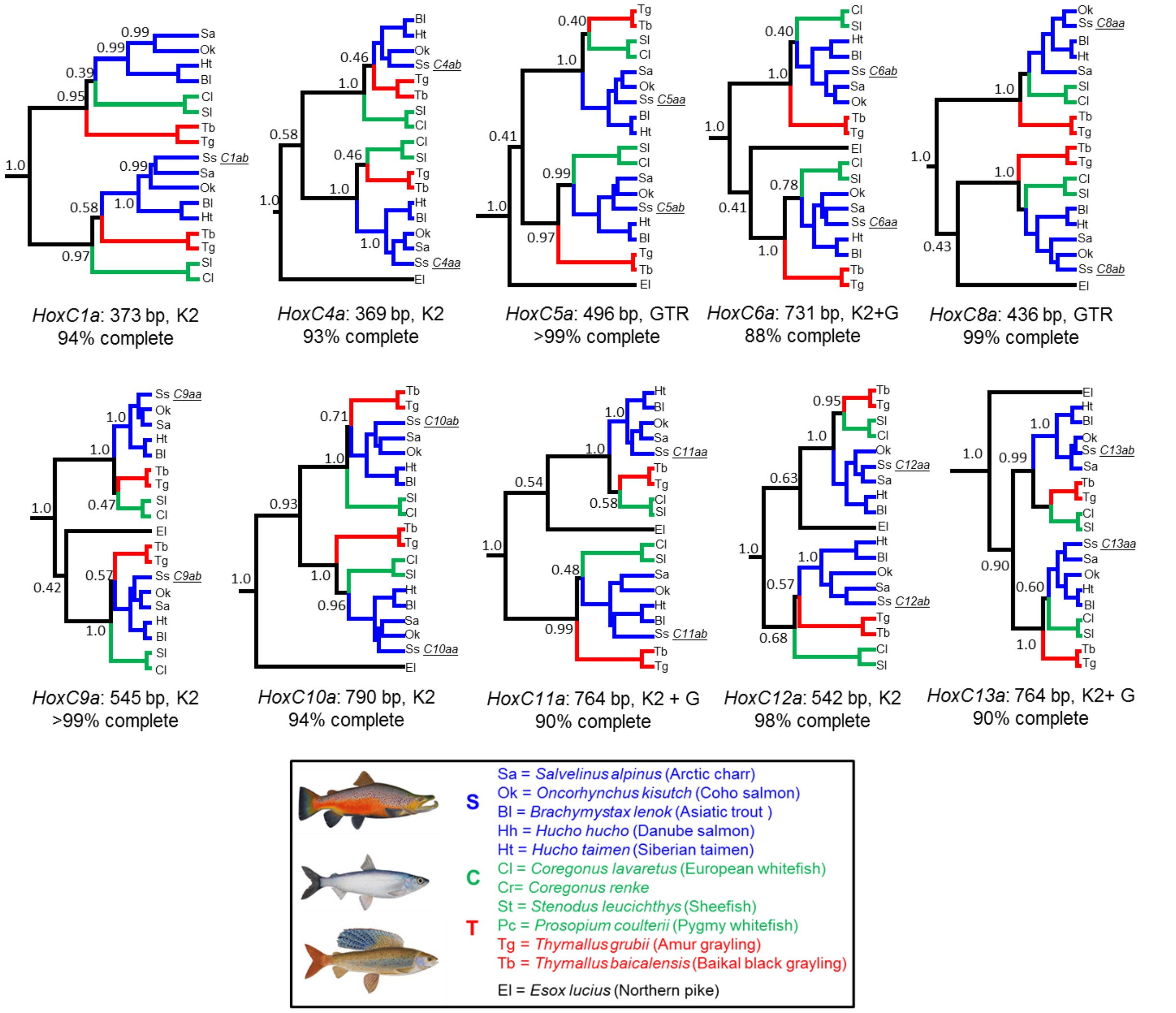

Fig. S6. Phylogenomic analysis of salmonid-specific HoxCa clusters. Individual Bayesian phylogenetic trees are shown for the sequence capture alignments combined for use in Fig. S9B. All other details are as provided in the Fig. S2 legend.

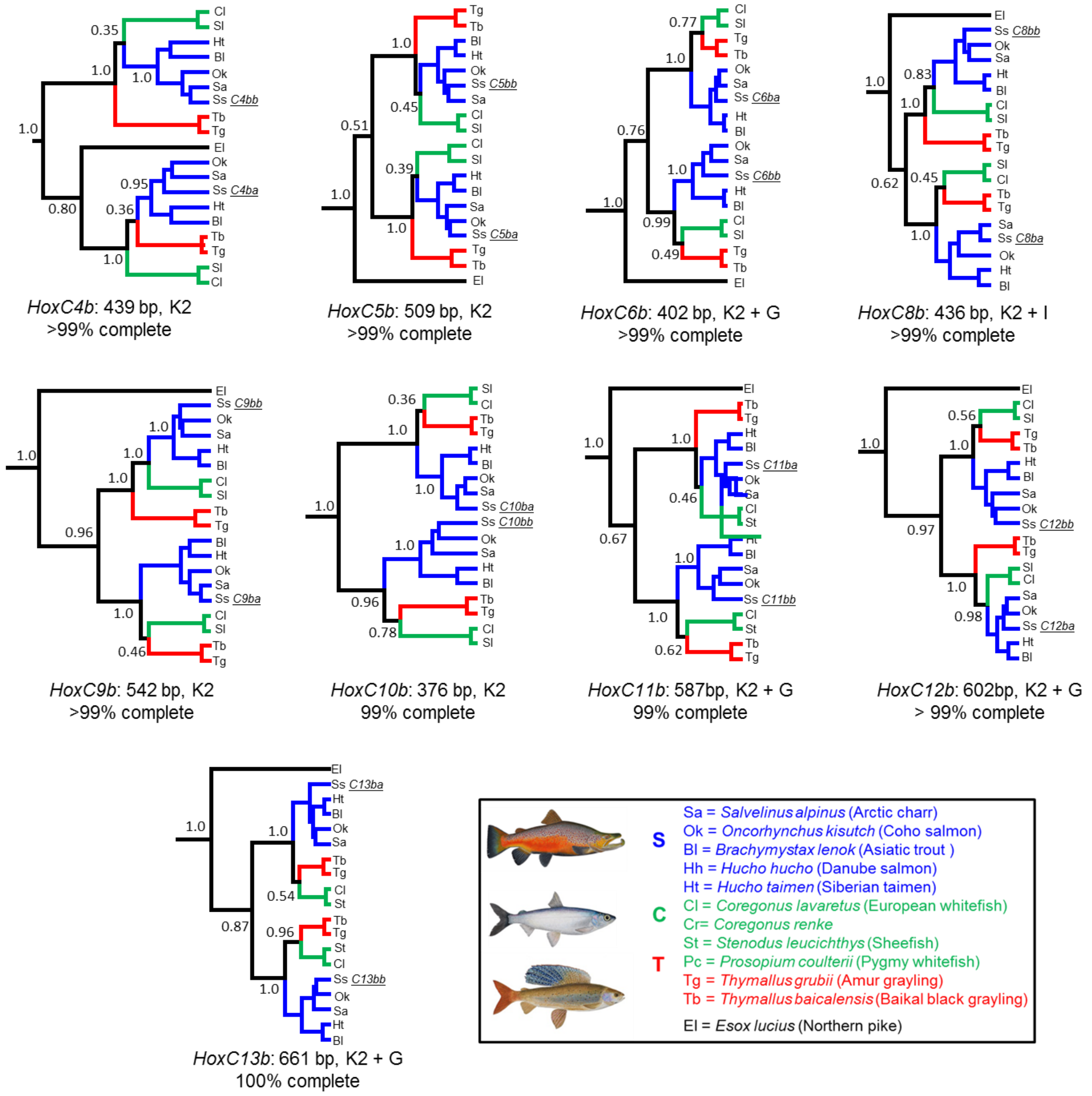

Fig. S7. Phylogenomic analysis of salmonid-specific HoxCb clusters. Individual Bayesian phylogenetic trees are shown for the sequence capture alignments combined for use in Fig. S9C. All other details are as provided in the Fig. S2 legend.

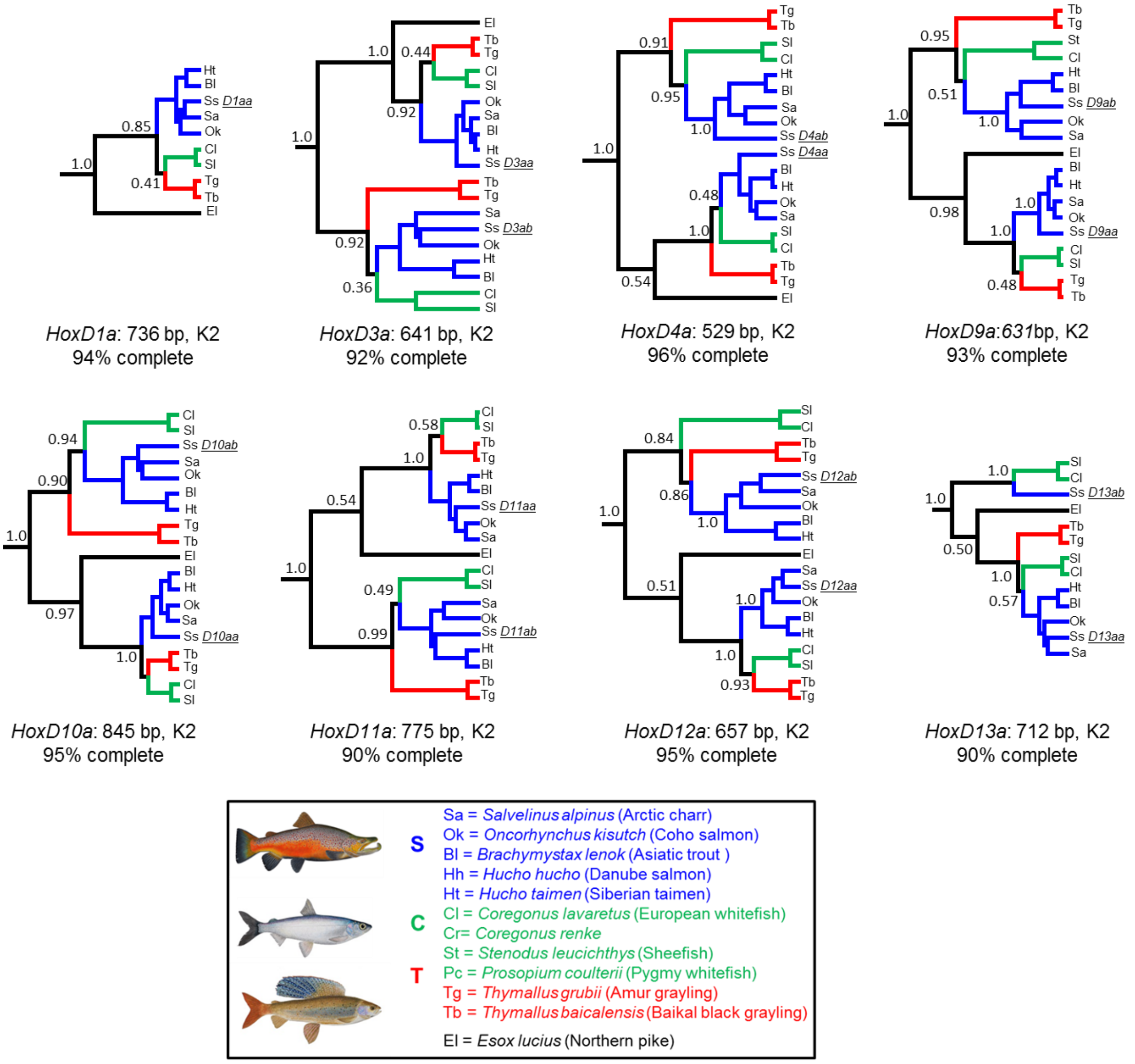

Fig. S8. **Phylogenomic analysis of salmonid-specific HoxDa clusters**. Individual Bayesian phylogenetic trees are shown for the sequence capture alignments combined for use in Fig. S9D. All other details are as provided in the Fig. S2 legend.

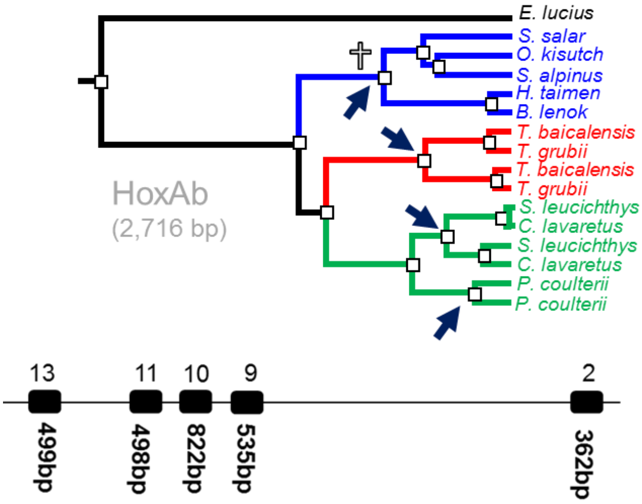

Fig. S9. The salmonid-specific HoxAb cluster conforms to predictions of LORe. Bayesian phylogenetic analysis are shown combining alignments along the HoxAb cluster. The colour of branches depict different salmonid subfamilies. The individual phylogenetic analyses combined for use in this figure are provided in Fig. S3. The cross depicts the putative loss of a Hox cluster in the common ancestor of Salmoninae. White boxes indicate posterior probability values >0.95. The organization of Hox clusters in Atlantic salmon [53] are shown below each tree, along with the length of alignments for individual Hox ohnologue pairs. Dark blue arrows highlight the inferred onset of ohnologue divergence.

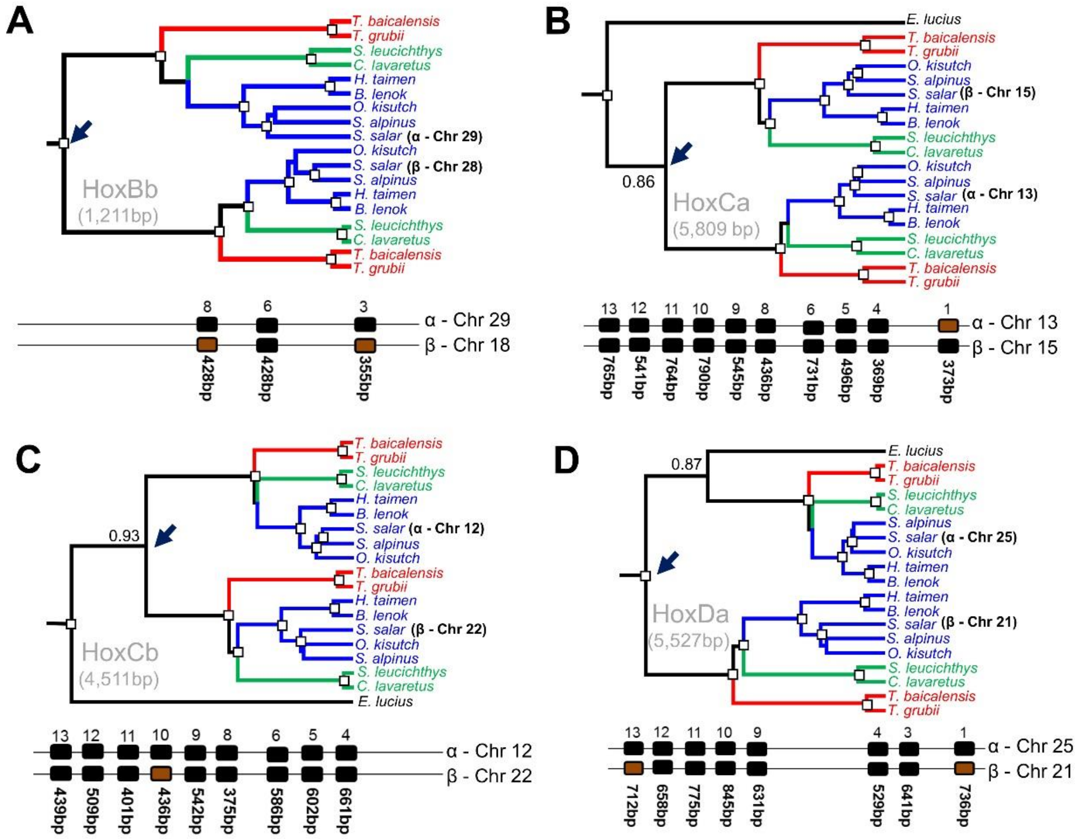

Fig. S10. Salmonid Hox clusters conforming to the AORe model. Bayesian phylogenetic analysis are shown combining alignments sampled along the HoxBb (**A**) HoxCa (**B**), HoxCb (**C**) and HoxDa (**D**) clusters. Individual phylogenetic analyses combined for use in this figure are provided in Fig. S5 (HoxBb), Fig. S6 (HoxCa), Fig. S7 (HoxCb) and Fig. S8 (HoxDa). For HoxBb, a pike outgroup to salmonid-specific WGD was not included in the analysis, as HoxB8b and HoxB3b orthologues were not identified in pike. Black-filled boxes represent functional genes and brown-filled boxes are putative pseudogenes (23). Other details are as provided in the Fig. S9 legend.

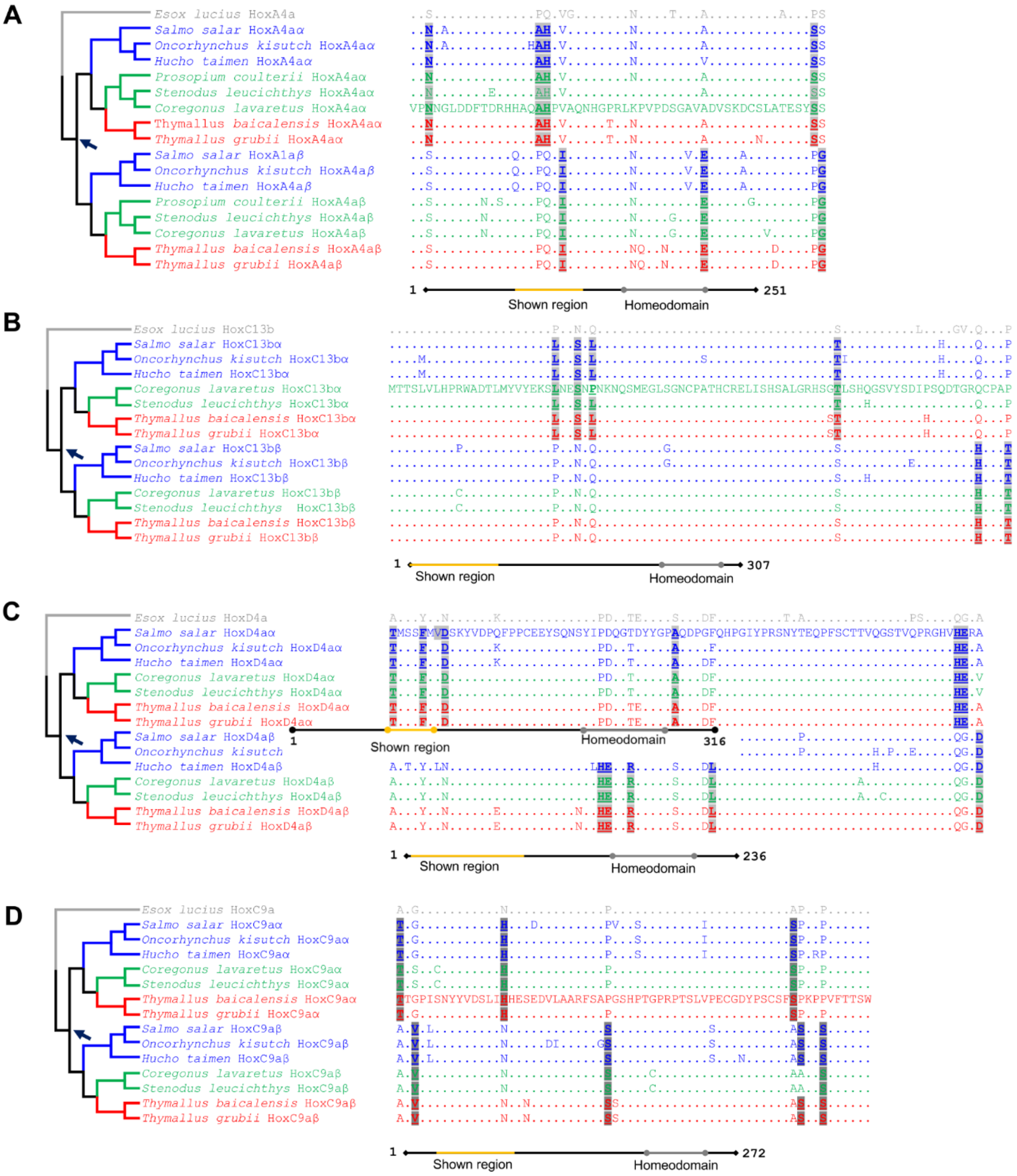

Fig. S11. Amino acid divergence between salmonid-specific Hox ohnologues under the AORe model for: (**A**) HoxA4a, (**B**) HoxC13b, (**C**) HoxD4a, and, (**D**) HoxC9a. Grey-shading highlights sites with fixed amino acid substitutions between ohnologues that arose in the salmonid ancestor and have been highly conserved. The amino acids directly highlighted are those that deviate from the ancestral state, informed by pike as an outgroup to the Ss4R WGD. Salmonid subfamilies are highlighted in different colours. The dark blue arrow on the trees depicts the onset of ohnologue divergence (rediploidization) in the salmonid ancestor. The region of the Hox protein shown is provided below each alignment, along with the location of the DNA-binding homeodomain.

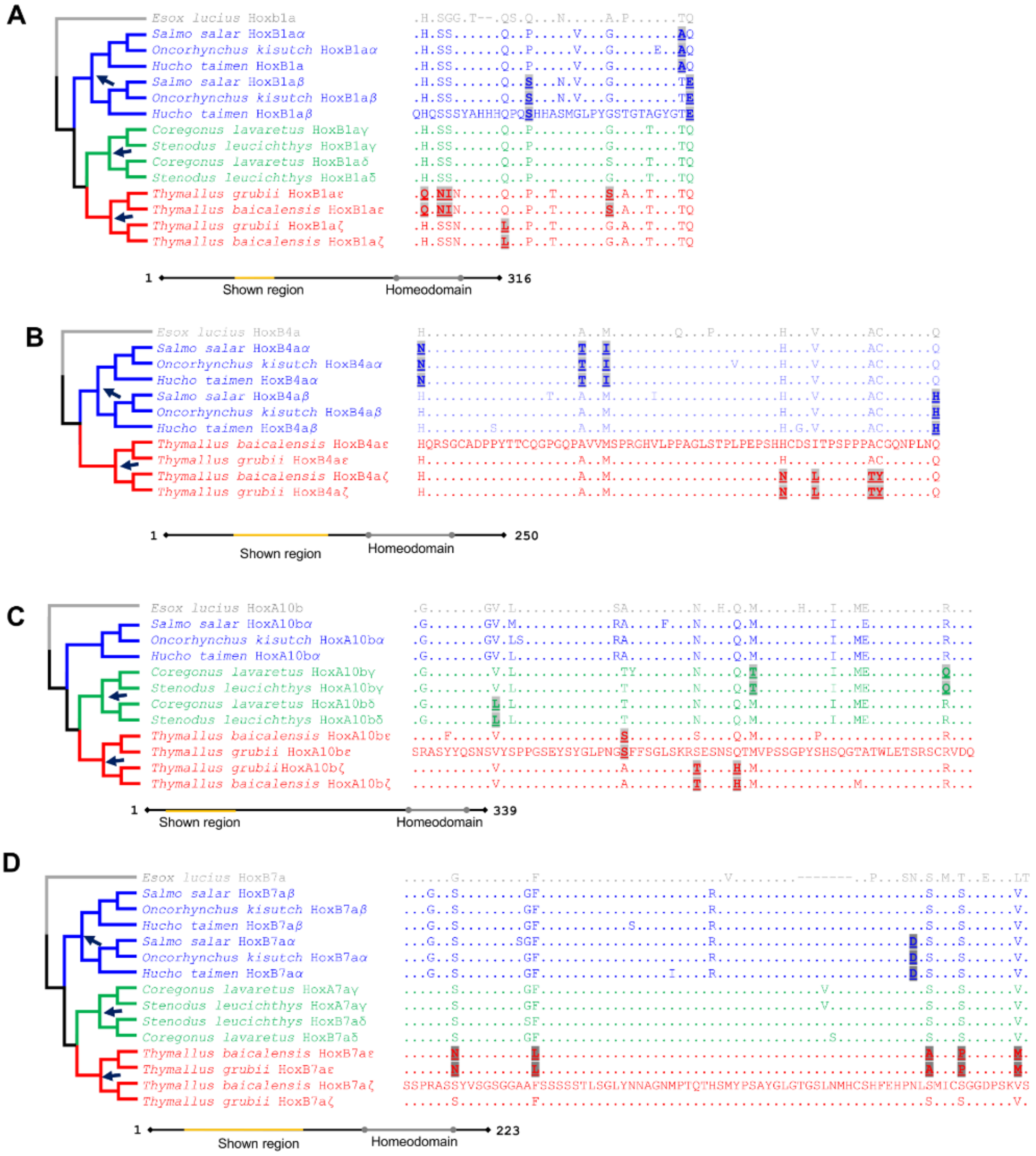

Fig. S12. Amino acid divergence between salmonid-specific ohnologues under the LORe model for: (**A**) HoxB1a, (**B**) HoxB4a, (**C**) HoxA10b, and, (**D**) HoxB7a. Grey-shading highlights sites where fixed differences evolved in ohnologues within different salmonid subfamilies and have subsequently been highly conserved. The actual sites highlighted are those that deviate from the ancestral state, informed with respect to pike as the outgroup to the Ss4R WGD outgroup. Other details are as provided in the Fig. S11 legend.

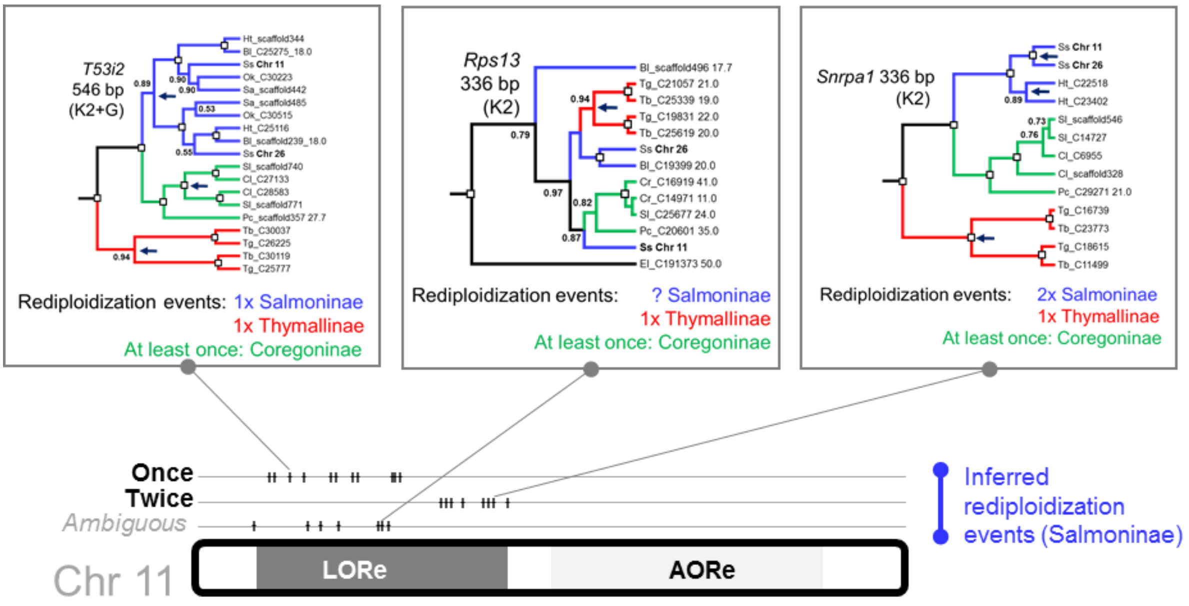

Fig. S13. Highlights another LORe region (distinct from example given in Fig. 5B) on Chr. 11 (paired with an equivalent region on Chr. 26; not shown, see Fig. 3), where the number of independent rediploidization events inferred within the Salmoninae is consistent along discrete regions of the genome. Example trees are shown for regions that evidently have distinct rediploidization histories. Species abbreviations: Ss: *S. salar*; Bl: *Brachymystax lenok*; Pc: *Prosopium coulterii*; Sl: *Stenodus leucichthys*; Cl: *Coregonus lavaretus*; Tb: *Thymallus baicalensis*; Tg: *Thymallus grubii*.

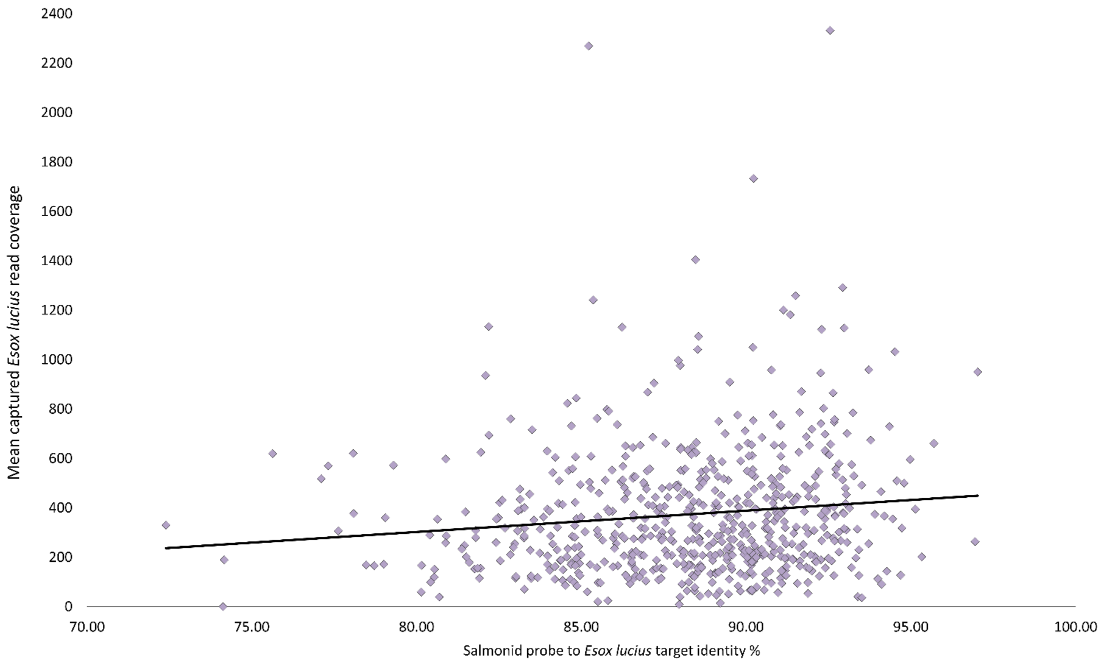

Fig. S14. Global efficiency of our sequence capture study. The plot shows the relationship between the mean number of northern pike reads captured (y-axis) in relation to the percentage identity shared between 'singleton' salmonid probe sequences (i.e. where only one salmonid ohnologue was present in the probe mix, even if more existed) and the equivalent target sequence in pike.

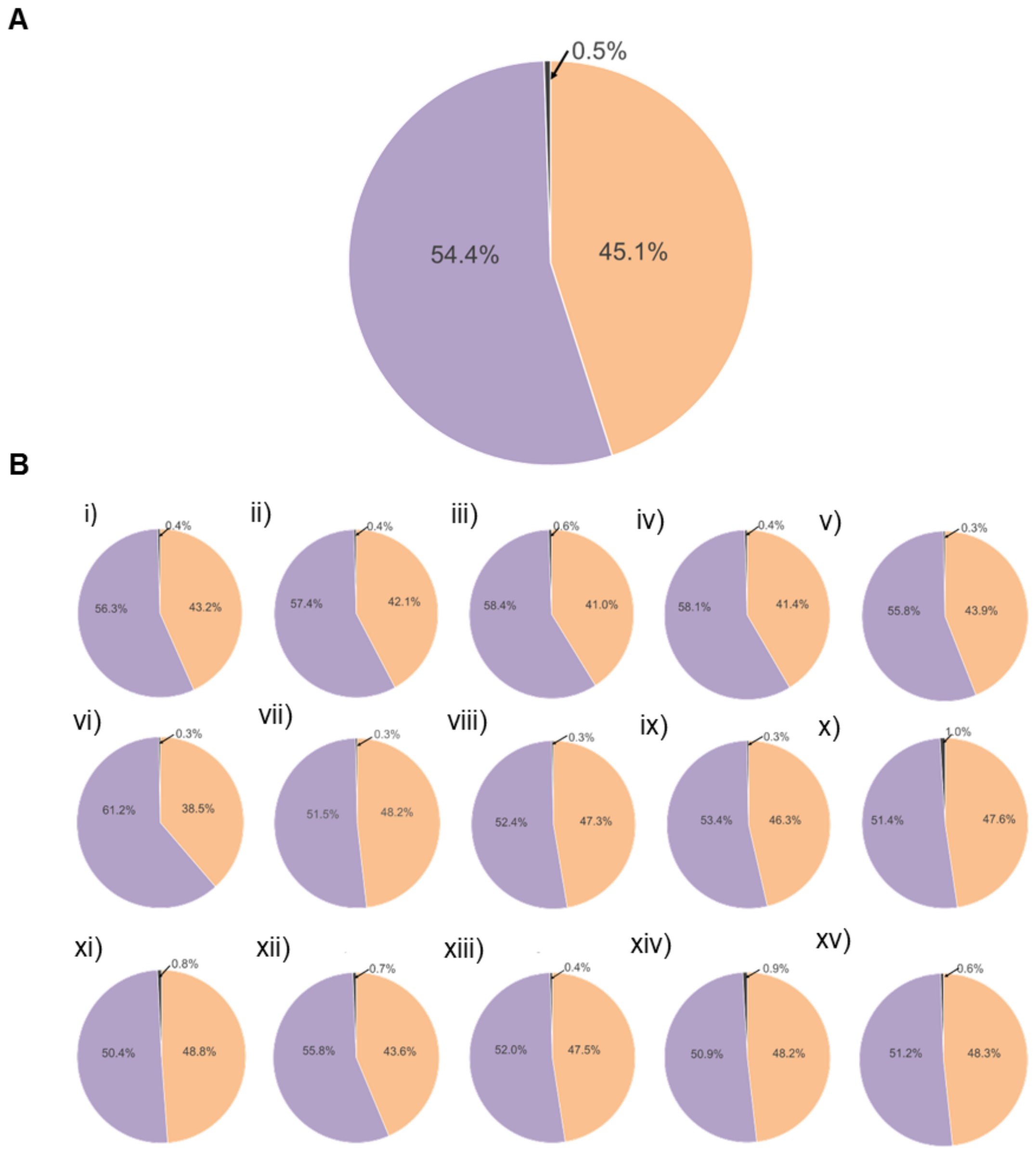

Fig. S15. Breakdown of BLAST analysis documenting the number of sequences recovered by the 893 singleton probes in the 15 targeted salmonid species. Orange segments indicate the capture of two paralogous sequences per singleton probe. Purple segments indicate the capture of only one gene. Black segments indicate no contigs recovered. (**A**) Average of all 15 species. (**B**) Individual data for all species, as follows; i) *O. kisutch*, ii) *O. nerka*, iii) *O. tshawytscha*, iv) *Salvelinus alpinus*, v) *Parahucho perryi*, vi) *S. trutta*, vii) *B. lenok*, viii) *Hucho hucho*, ix) *H. taimen*, x) *Thymallus baicalensis*, xi) *T. grubii*, xii) *Prosopium coulteri*, xiii) *C. lavaretus*, xiv) *C. renke*, xv) *Stenodus leucichthys*.

Table S1. Candidate Atlantic salmon ohnologues implicated in the evolution of anadromous life-history

Group	Function	Model	Short name	Full name	Ohnologue 1 accession	Ohnologue 1 location	Ohnologue 2 accession	Ohnologue 2 location	Pearson correlation
IGF-GH	IGF-GH signaling	LORe	*GH*	Somatotropin / growth hormone	XM_014204437.1	ssa06	NM_001123676.1	ssa03	N/A
IGF-GH	IGF-GH signaling	AORe	*GHR*	Growth hormone receptor isoform 2 precursor	NM_001123594.1	ssa11	XM_014133881.1	ssa01	0.51
IGF-GH	IGF-GH signaling	LORe	*IGF1*	Insulin-like growth factor I	XM_014208342.1	ssa07	XM_014153327.1	ssa17	0.74
IGF-GH	IGF-GH signaling	AORe	*IGFBP1B*	IGF binding protein 1 (teleost paralogue B)	NM_001123624.1	ssa14	NM_001279165.1	ssa03	N/A
IGF-GH	IGF-GH signaling	LORe	*IGFBP2A*	IGF binding protein 2 (teleost paralogue A)	NM_001123625.1	ssa16	XM_014152142.1	ssa17	0.85
IGF-GH	IGF-GH signaling	AORe	*IGFBP3B*	Insulin-like growth factor binding protein 3 (teleost paralogue B)	NM_001279167.1	ssa14	XM_014190936.1	ssa03	N/A
IGF-GH	IGF-GH signaling	LORe	*IGFBP4*	Insulin-like growth factor-binding protein 4	XM_014131503.1	ssa12	NM_001139586.1	ssa02	0.68
IGF-GH	IGF-GH signaling	LORe	*IGFBP5A*	Insulin-like growth factor-binding protein 5 (teleost paralogue A)	XM_014150691.1	ssa16	XM_014152143.1	ssa17	0.82
IGF-GH	IGF-GH signaling	AORe	*IGFBP5B*	Insulin-like growth factor binding protein 5 (teleost paralogue B)	NM_001279142.1	ssa21	NM_001123649.1	ssa25	-0.06
IGF-GH	IGF-GH signaling	AORe	*IGFBP6A*	Insulin-like growth factor binding protein 6 (teleost paralogue A)	NM_001279145.1	ssa12	XM_014167271.1	ssa22	N/A
IGF-GH	IGF-GH signaling	AORe	*IGFBP6B*	Insulin-like growth factor binding protein 6 (teleost paralogue B)	XM_014135597.1	ssa13	NM_001123650.1	ssa15	0.47
IGF-GH	IGF-GH signaling	AORe	*IGFR*	Insulin-like growth factor 1 receptor	XM_014124119.1	ssa10	XM_014149105.1	ssa16	0.90
THR	Thyroid signaling	LORe	*THRA1*	Thyroid hormone receptor alpha-like	XM_014168488.1	ssa23	XM_014192391.1	ssa03	-0.95
THR	Thyroid signaling	AORe	*THRA2*	Thyroid hormone receptor alpha-like	XM_014159154.1	ssa19	XM_014179098.1	ssa28	0.59
THR	Thyroid signaling	AORe	*THRB*	Thyroid hormone receptor, beta	XM_014142418.1	ssa14	XM_014177879.1	ssa27	0.68
THR	Thyroid signaling	AORe	*TRH*	Thyrotropin-releasing hormone	NM_001141172.1	ssa13	XM_014145411.1	ssa15	N/A

GLUC	Glucocorticoid signaling	AORe	*GR*	Glucocorticoid receptor	XM_014198059.1	ssa04	XM_014136782.1	ssa13	0.26
GLUC	Glucocorticoid signaling	LORe	*MR*	Mineralocorticoid receptor-like	XM_014209388.1	ssa08	XM_014194959.1	ssa04	0.68
CA	Acid-base balance	AORe	*CA*	Carbonic anhydrase	NM_001140297.1	ssa14	XM_014190532.1	ssa03	-0.51
CA	Acid-base balance	LORe	*CA10a*	Carbonic anhydrase-related protein 10-like	XM_014203883.1	ssa06	XM_014193344.1	ssa03	1.00
CA	Acid-base balance	AORe	*CA10b*	Carbonic anhydrase-related protein 10	XM_014158122.1	ssa19	XM_014179440.1	ssa28	N/A
CA	Acid-base balance	AORe	*CA5a*	Carbonic anhydrase VA, mitochondrial	XM_014124398.1	ssa10	XM_014148864.1	ssa16	-0.01
CA	Acid-base balance	LORe	*CA7*	Carbonic anhydrase 7-like	XM_014126475.1	ssa11	XM_014175514.1	ssa26	N/A
CA	Acid-base balance	AORe	*CA8*	Carbonic anhydrase-related protein-like	XM_014139629.1	ssa14	XM_014190871.1	ssa03	1.00
CLC	Cl ion transport	AORe	*CLCN1*	Chloride channel protein 1-like	XM_014141823.1	ssa14	XM_014177430.1	ssa27	1.00
CLC	Cl ion transport	AORe	*CLCN2*	Chloride channel protein 2-like	XM_014139596.1	ssa14	XM_014190851.1	ssa03	N/A
CLC	Cl ion transport	AORe	*CLCN3*	Chloride channel 3	XM_014206955.1	ssa07	XM_014156271.1	ssa18	0.35
CLC	Cl ion transport	LORe	*CLCN4*	H(+)/Cl(-) exchange transporter 4	XM_014150020.1	ssa16	XM_014151496.1	ssa17	0.48
CLC	Cl ion transport	AORe	*CLCN5*	H(+)/Cl(-) exchange transporter 5-like	XM_014129474.1	ssa11	XM_014196009.1	ssa04	0.48
CLC	Cl ion transport	AORe	*CLIC1*	Chloride intracellular channel protein 1-like	XM_014142014.1	ssa14	XM_014176986.1	ssa27	0.44
CLC	Cl ion transport	AORe	*CLIC2*	Chloride intracellular channel 2	XM_014199024.1	ssa05	XM_014211812.1	ssa09	-0.22
NKAIN	Interacts with N+/K+ pump	AORe	*NKAIN1*	Sodium/potassium-transporting ATPase subunit beta-1-interacting protein 1-like	XM_014143057.1	ssa14	XM_014178117.1	ssa27	0.65
NKAIN	Interacts with N+/K+ pump	AORe	*NKAIN2*	Na+/K+ transporting ATPase interacting 2	XM_014205110.1	ssa06	XM_014144382.1	ssa15	0.90
NKAIN	Interacts with N+/K+ pump	AORe	*NKAIN3*	Sodium/potassium-transporting ATPase subunit beta-1-interacting protein 3-like	XM_014136253.1	ssa13	XM_014146658.1	ssa15	1.00

SLC	Na+/Cl- transport	AORe	*SLC12A2*	solute carrier family 12 member 2-like	XM_014127974.1	ssa11	NM_001123683.1	ssa01	0.02
SLC	K+/Cl- transport	LORe	*SLC12A4*	solute carrier family 12 member 4-like	XM_014127285.1	ssa11	XM_014176164.1	ssa26	0.71
SLC	K-Cl cotransport	AORe	*SLC12A5a*	solute carrier family 12 member 5-like	XM_014134883.1	ssa13	XM_014145359.1	ssa15	N/A
SLC	K-Cl cotransport	AORe	*SLC12A5b*	solute carrier family 12 member 5-like	XM_014131710.1	ssa12	XM_014168172.1	ssa22	N/A
SLC	K-Cl cotransport	AORe	*SLC12A7*	solute carrier family 12 member 7-like	XM_014140329.1	ssa14	XM_014190430.1	ssa03	0.47
SLC	Electroneutral cation-Cl cotransport	AORe	*SLC12A9a*	solute carrier family 12 member 9-like	XM_014137731.1	ssa13	XM_014197472.1	ssa04	0.74
SLC	Na+/(Ca2+ K+) exchange	AORe	*SLC24A2*	sodium/potassium/calcium exchanger 2-like	XM_014207460.1	ssa07	XM_014155447.1	ssa18	N/A
SLC	Na+/(Ca2+ K+) exchange	LORe	*SLC24A3a*	sodium/potassium/calcium exchanger 3-like	XM_014130968.1	ssa12	XM_014173839.1	ssa02	N/A
SLC	Na+/(Ca2+ K+) exchange	AORe	*SLC24A3b*	sodium/potassium/calcium exchanger 3-like	XM_014203625.1	ssa01	XM_014154585.1	ssa18	1.00
SLC	Multifunctional anion exchange	AORe	*SLC24A4*	sodium/potassium/calcium exchanger 4-like	XM_014206017.1	ssa06	XM_014144976.1	ssa15	N/A
SLC	Multifunctional anion exchange	AORe	*SLC26A2*	Sulfate transporter-like	XM_014129156.1	ssa01	XM_014138169.1	ssa13	0.01
SLC	Multifunctional anion exchange	LORe	*SLC26A3*	Chloride anion exchanger-like	XM_014208144.1	ssa07	XM_014153307.1	ssa17	-0.75
SLC	Multifunctional anion exchange	LORe	*SLC26A4*	Pendrin-like	XM_014208140.1	ssa07	XM_014153304.1	ssa17	1.00
SLC	Multifunctional anion exchange	LORe	*SLC26A5*	Prestin-like	XM_014208788.1	ssa07	XM_014153721.1	ssa17	0.41
SLC	Bicarbonate transport	LORe	*SLC4A1*	Band 3 anion exchange protein-like	XM_014203255.1	ssa06	XM_014194002.1	ssa03	0.93
SLC	Bicarbonate transport	LORe	*SLC4A10a*	Sodium-driven chloride bicarbonate exchanger-like	XM_014150253.1	ssa16	XM_014151732.1	ssa17	1.00
SLC	Bicarbonate transport	AORe	*SLC4A10b*	Sodium-driven chloride bicarbonate exchanger-like	XM_014164135.1	ssa21	XM_014173157.1	ssa25	1.00
SLC	Bicarbonate transport	AORe	*SLC4A2*	Anion exchange protein 2-like	XM_014157742.1	ssa19	XM_014180860.1	ssa29	-0.29
SLC	Bicarbonate transport	AORe	*SLC4A3*	Anion exchange protein 3-like	XM_014164421.1	ssa21	XM_014174515.1	ssa25	1.00

SLC	Bicarbonate transport	AORe	*SLC4A7*	Sodium bicarbonate cotransporter 3-like	XM_014142039.1	ssa14	XM_014176946.1	ssa27	0.68
SLC	Na+/Ca2+ exchange	AORe	*SLC8A1*	Sodium/calcium exchanger 1-like	XM_014206223.1	ssa01	XM_014154971.1	ssa18	0.99
SLC	Na+/H+ exchange	LORe	*SLC9A1*	Sodium/hydrogen exchanger 1-like	XM_014200499.1	ssa05	XM_014166485.1	ssa02	0.90
SLC	Na+/H+ exchange	AORe	*SLC9A6*	Sodium/hydrogen exchanger 6-like	XM_014199742.1	ssa05	XM_014212219.1	ssa09	0.28
SLC	Na+/H+ exchange	LORe	*SLC9B2*	Mitochondrial sodium/hydrogen exchanger 9B2-like	XM_014209140.1	ssa08	XM_014195592.1	ssa04	0.25

N/A – Pearson’s correlation not possible (expression limited to too few tissues)

Table S2. Details of the genomic locations and clustering index (see Methods) for LORe ohnologue pairs contributing to significantly enriched GO terms (see Additional file 4 for full data)

GO.ID	Term	Ohnologue pairs span the following duplicated regions:	Clustering index
GO:0046219	Indolalkylamine biosynthetic process	Ssa02-05 (n=1); Ssa03-06 (n=2); Ssa07-17 (n=2); Ssa11-26 (n=1)	0.50
GO:0006586	Indolalkylamine metabolic process	Ssa02-12 (n=1); Ssa03-06 (n=3); Ssa04-08 (n=1); Ssa07-17 (n=2); Ssa02-05 (n=1); Ssa16-17 (n=1); Ssa11-26 (n=1)	0.20
GO:0090403	Oxidative stress-induced premature senescence	Ssa07-17 (n=2); Ssa11-26 (n=1); Ssa16-17 (n=1)	0.00
GO:0031033	Myosin filament organization	Ssa02-05 (n=2); Ssa02-12 (n=4); Ssa03-06 (n=4); Ssa03-23 (n=1) ; Ssa11-26 (n=2); Ssa07-17 (n=1); Ssa16-17 (n=1)	0.23
GO:1903319	Positive regulation of protein maturation	Ssa02-05 (n=2); Ssa02-12 (n=4); Ssa03-06 (n=2); Ssa07-17 (n=1); Ssa11-26 (n=2); Ssa16-17 (n=1)	0.09
GO:0031034	Myosin filament assembly	Ssa02-05 (n=1); Ssa02-12 (n=4); Ssa03-06 (n=3); Ssa03-23 (n=1); Ssa07-17 (n=1); Ssa11-26 (n=2)	0.21
GO:0034769	Basement membrane disassembly	Ssa02-05 (n=2); Ssa02-12 (n=1); Ssa07-17 (n=1)	0.25
GO:0042274	Ribosomal small subunit biogenesis	Ssa02-05 (n=5); Ssa02-12 (n=4); Ssa03-06 (n=6); Ssa07-17 (n=1); Ssa11-26 (n=2); Ssa16-17 (n=1)	0.11
GO:0015669	Gas transport	Ssa02-12 (n=1); Ssa03-06 (n=7); Ssa11-26 (n=1)	0.53
GO:0038202	TORC1 signalling	Ssa02-12 (n=1); Ssa03-06 (n=2)	0.00
GO:0071681	Cellular response to indole-3-methanol	Ssa02-12 (n=1); Ssa03-06 (n=1); Ssa04-08 (n=1); Ssa11-26 (n=1)	0.00
GO:1903918	Regulation of actin filament severing	Ssa02-12 (n=3); Ssa03-06 (n=1)	0.28
GO:0042435	Indole-containing compound biosynthetic process	Ssa02-05 (n=1); Ssa03-06 (n=2); Ssa04-08 (n=1); Ssa07-17 (n=2); Ssa11-26 (n=1)	0.30
GO:0009407	Toxin catabolic process	Ssa02-05 (n=3); Ssa02-12 (n=1); Ssa11-26 (n=2); Ssa16-17 (n=1)	0.29
GO:0090487	Secondary metabolite catabolic process	Ssa02-05 (n=3); Ssa02-12 (n=1); Ssa11-26 (n=2); Ssa16-17 (n=1)	0.20
GO:0010728	Regulation of hydrogen peroxide biosynthetic process	Ssa02-12 (n=1); Ssa03-06 (n=1); Ssa04-08 (n=6)	0.66
GO:0010729	Positive regulation of hydrogen peroxide biosynthetic process	Ssa04-08 (n=6)	0.73
GO:0030421	Defecation	Ssa03-06 (n=2); Ssa11-26 (n=3) ; Ssa16-17 (n=1)	0.00
GO:0071260	Cellular response to mechanical stimulus	Ssa02-05 (n=4); Ssa02-12 (n=4); Ssa03-06 (n=7); Ssa03-23 (n=1); Ssa04-08 (n=2); Ssa05-09 (n=1); Ssa07-17 (n=5); Ssa16-17 (n=3); Ssa11-26 (n=3)	0.14
GO:0015671	Oxygen transport	Ssa02-12 (n=1) ; Ssa03-06 (n=5)	0.70

Table S3. Statistics for sequence capture assemblies across 16 study species

Latin name	Common name	Captured reads	Reads post-QC	Number Contigs	Largest contig (bp)	Sum contig length (bp)	GC (%)	Contig N50	N's per 100 kb
Salvelinus alpinus	Arctic charr	22,463,636	22,257,292	13,838	6,239	62,541,62	46.04	936	1,678.42
Oncorhynchus kisutch	Coho salmon	22,677,672	22,469,070	13,040	8,533	62,904,76	45.96	970	1,649.08
Oncorhynchus nerka	Sockeye salmon	21,883,220	21,697,420	13,447	9,231	62,480,44	46.09	948	1,619.46
Oncorhynchus tshawytscha	Chinook salmon	21,692,844	21,497,517	13,088	7,484	59,846,57	46.13	914	2,016.37
Parahucho perryi	Sakhalin taimen	22,858,324	22,665,538	12,324	9,035	62,897,37	46.08	944	1,369.68
Salmo trutta	Brown trout	23,400,536	23,197,420	13,569	5,385	60,509,54	46.24	889	2,028.92
Brachymystax lenok	Sharp-snouted lenok	22,350,830	22,149,040	11,512	9,918	62,119,97	46.03	937	1,252.61
Hucho hucho	Danube salmon	22,206,534	22,149,040	12,336	8,912	61,674,01	46.19	921	1,522.87
Hucho taimen	Siberian taimen	20,231,922	20,054,918	11,582	8,686	57,140,37	46.42	912	1,662.28
Prosopium coulterii	Pygmy whitefish	21,972,906	21,770,351	12,882	8,968	57,507,12	46.60	890	1,742.62
Coregonus lavaretus	European whitefish	22,647,902	22,424,151	13,177	7,316	56,200,95	46.68	854	1,991.27
Coregonus renke	Whitefish spp.	21,368,428	21,175,075	13,651	7,386	56,552,55	46.59	859	2,205.31
Stenodus leucichthys	Sheefish spp.	21,443,062	21,251,058	12,901	10,083	59,589,23	46.32	909	1,659.18
Thymallus baicalensis	Baikal black grayling	21,911,578	21,705,293	12,807	9,159	59,643,20	46.10	906	1,584.04
Thymallus grubii	Amur grayling	20,738,450	20,550,750	11,592	10,486	57,855,48	46.19	915	1,319.36
Esox lucius	Northern pike	20,885,504	20,695,801	59,752	5,699	180,551,77	45.71	716	1,062.71
