# Supplementary figures and images for: Lineage-specific rediploidization is a mechanism to explain time-lags between genome duplication and evolutionary diversification
Source: Genome Biol. 2017 Jun 14;18:111. doi: 10.1186/s13059-017-1241-z (PMC5470254; doi:10.1186/s13059-017-1241-z)

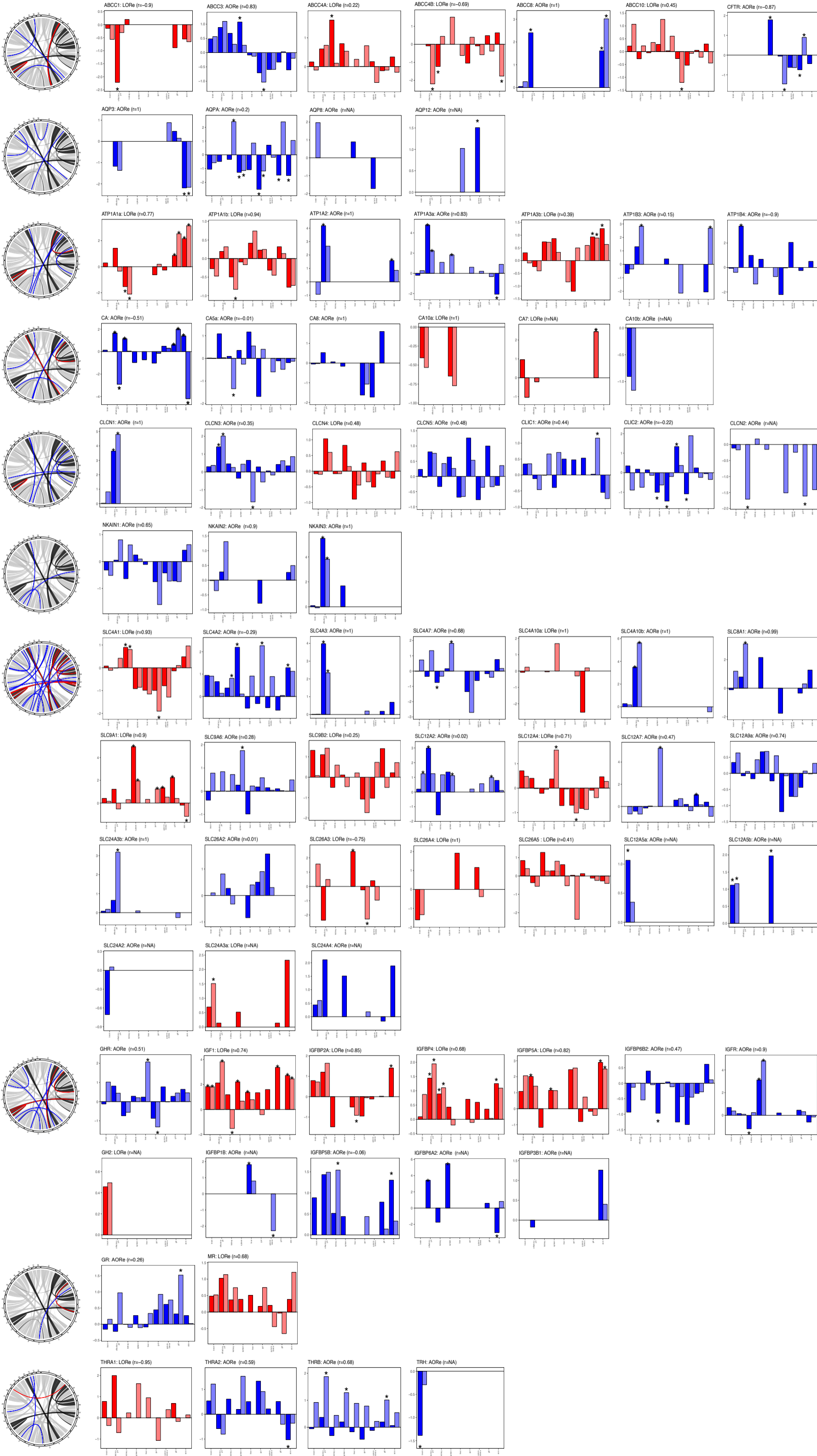

Supplement: Supplementary file 3 — Full ohnologue expression response data (summarized in Fig. 7). Multi-tissue expression responses accompanying the freshwater to saltwater transition for candidate Atlantic salmon ohnologues with implied functions in smoltification and anadromous life-history. (PDF 1844 kb) [file 13059_2017_1241_MOESM3_ESM.pdf]
